# A survey of linyphiid spiders (Arachnida, Linyphiidae) from Guiyang, Guizhou Province, China, with description of a new species

**DOI:** 10.3897/BDJ.14.e210136

**Published:** 2026-09-16

**Authors:** Wanting Zhao, Lianfeng He, Xiaolin Chen, Jianshuang Zhang, Hao Yu

**Affiliations:** 1 The State Key Laboratory of Southwest Karst Mountain Biodiversity Conservation of Forestry Administration, School of Life Sciences, Guizhou Normal University, Guiyang, Guizhou, China, Guiyang, China The State Key Laboratory of Southwest Karst Mountain Biodiversity Conservation of Forestry Administration, School of Life Sciences, Guizhou Normal University, Guiyang, Guizhou, China Guiyang China

**Keywords:** biodiversity, fauna, hammock-web spiders, new record, taxonomy

## Abstract

**Background:**

Linyphiidae Blackwall, 1859 is the second-largest spider family, currently comprising nearly 5,000 species worldwide. Although Guiyang is one of the most biodiverse provincial capitals in China, its linyphiid fauna remains poorly known, with only a single species previously recorded.

**New information:**

Recent field surveys conducted by the authors in Guiyang revealed 17 species of Linyphiidae, including one new to science. We provide the first checklist of the family from Guiyang, together with a distribution map of all recorded species and live habitus photographs for 13 species (the remaining four species were not photographed in the field). A detailed description and diagnostic micrographs of the new species are presented and a partial mitochondrial cytochrome c oxidase subunit I (COI) sequence is provided to facilitate future molecular studies.

## Introduction

The hammock-web spider family Linyphiidae Blackwall, 1859 is the second-largest family in the order Araneae, currently comprising 641 extant genera and 4,976 species distributed worldwide, except Antarctica. Of these, 186 genera and 627 species have been recorded from China (World Spider Catalog 2026, Shi et al. 2026).

Linyphiid spiders have traditionally been considered most diverse in the temperate and cold regions of the Northern Hemisphere, with substantially lower diversity in tropical and subtropical areas (Sørensen et al. 2002, Floren and Deeleman-Reinhold 2005, Jocqué and Dippenaar-Schoeman 2006, Zhao and Li 2014. However, recent comprehensive surveys have demonstrated that linyphiids are also highly diverse and abundant in the tropical and subtropical parts of the Oriental Region (Zhao and Li 2014, Irfan 2019, Irfan et al. 2022, Irfan et al. 2025).

Guiyang, the provincial capital of Guizhou Province (Fig. 1A), was the first city in China to be awarded the title of “National Forest City” and is renowned for its subtropical forests, which cover more than 55% of its land area (Zhong 2018). The city is also known as the “City of a Thousand Gardens”, with more than 1,000 urban parks and is regarded as one of the most biodiverse provincial capitals in China (Yang 2020, Cheng and Chen 2022). Despite this high level of biodiversity, the spider fauna of Guiyang remains poorly documented. To date, only 67 species belonging to 26 families have been recorded or described from the city (HY, unpublished data). Amongst these, 23 species are endemic, 18 were originally described from Guiyang and 17 have been reported as new records in recent years (Zhou et al. 2026). Nevertheless, the known spider diversity of Guiyang almost certainly represents only a fraction of its actual fauna.

The family Linyphiidae is particularly poorly studied in Guiyang. Prior to this study, only a single species, *Parameioneta bilobata* Li & Zhu, 1993, had been recorded from the city (Li and Zhu 1995). Here, we report the results of the first comprehensive survey of linyphiid spiders in Guiyang, documenting 17 species, including 16 previously known species and one species new to science (Fig. 1B, Figs 2, 3, 4, 5). Accordingly, this study aims to: (1) describe *Lenis guiyang* He, Yu & Zhang, sp. nov. and provide its DNA barcode; (2) compile the first checklist of the linyphiid spiders of Guiyang, with photographs of live specimens whenever available; and (3) map the distribution of all recorded linyphiid species in Guiyang City.

## Materials and methods

Specimens were primarily collected by beating and searching. All examined materials are deposited in The State Key Laboratory of Southwest Karst Mountain Biodiversity Conservation of Forestry Administration, Guizhou Normal University (**GZNU**) in Guiyang, China.

Specimens were examined with an Olympus SZX7 stereomicroscope; details were studied with an Olympus BX41 compound microscope. Male palps and female epigynes were examined and illustrated after being dissected. Epigynes were removed and cleared in warm lactic acid before illustration. Vulvae were also imaged after being embedded in Arabic gum. Photos were made with a Canon EOS70D digital camera mounted on an Olympus CX41 compound microscope. The digital images were taken and assembled using Helicon Focus ver. 3.10.3. software package (Khmelik et al. 2005). The distribution maps were generated with ArcGis ver.10.5 (ESRI Inc 2002).

A partial fragment of the mitochondrial cytochrome oxidase subunit I (COI) gene was amplified and sequenced for a specimen of *L. guiyang* sp. nov., using the primers LCO1490 (5’-GGTCAACAAATCATAAAGATATTG-3’) and HCO2198 (5’-TAAACTTCAGGGTGACCAAAAAAT-3’) (Folmer et al. 1994). For additional information on extraction, amplification and sequencing procedures, see Wheeler et al. (2017). Sequence was trimmed to 633 bp, confirmed using BLAST and is deposited in GenBank. The code and GenBank accession numbers of the voucher specimen are provided as follows: *L. guiyang* sp. nov: YHGY215, ♀, GenBank PZ944867.

All measurements were obtained using an Olympus SZX7 stereomicroscope and given in millimetres. Eye diameters are taken at widest point. The total body length does not include chelicerae or spinnerets length. Leg lengths are given as total length (femur, patella, tibia, metatarsus, tarsus). References to figures in the cited papers are listed in lowercase (fig. or figs.); figures from this paper are noted with an initial capital (Fig. or Figs). The terminology used in text and figure legends follows Irfan et al. (2022), Irfan et al. (2025).

## Taxon treatments

### Lenis guiyang

He, Yu & Zhang sp. nov.

4C35BF00-151B-5A0C-8C82-0E0895316F27

1F995335-25C2-479B-9380-94933107FF15

#### Materials

- **Type status:** Holotype. **Occurrence:** recordedBy: Qianle Lu; individualCount: 1; sex: female; lifeStage: adult; occurrenceID: A485B528-B4C3-5E2E-832F-77224B3478D7; **Taxon:** scientificName: *Lenis guiyang*; order: Araneae; family: Linyphiidae; genus: *Lenis*; specificEpithet: *guiyang*; scientificNameAuthorship: He, Yu & Zhang; **Location:** continent: Asia; country: China; countryCode: CHN; stateProvince: Guizhou; county: Xifeng; municipality: Guiyang; locality: Mt. Xiwang, Fengchi Temple; verbatimElevation: 1437 m; verbatimCoordinates: 27.13°N, 106.64°E; decimalLatitude: 27.13; decimalLongitude: 106.64; georeferenceProtocol: label; **Identification:** identifiedBy: Hao Yu; dateIdentified: 2026; **Event:** samplingProtocol: beating; samplingEffort: 10 km by foot; eventDate: 6/6/2022; **Record Level:** language: en; basisOfRecord: PreservedSpecimen; **Material Entity:** preparations: whole animal (ETOH); associatedSequences: Specimen code: YHGY215; GenBank: PZ944867
- **Type status:** Paratype. **Occurrence:** recordedBy: Qianle Lu; individualCount: 1; sex: female; lifeStage: adult; occurrenceID: D8522714-559B-5AE2-825D-5BF85AE1E61A; **Taxon:** scientificName: *Lenis guiyang*; order: Araneae; family: Linyphiidae; genus: *Lenis*; specificEpithet: *guiyang*; scientificNameAuthorship: He, Yu & Zhang; **Location:** continent: Asia; country: China; countryCode: CHN; stateProvince: Guizhou; county: Xifeng; municipality: Guiyang; locality: Mt. Xiwang, Fengchi temple; verbatimElevation: 1437 m; verbatimCoordinates: 27.13°N, 106.64°E; decimalLatitude: 27.13; decimalLongitude: 106.64; georeferenceProtocol: label; **Identification:** identifiedBy: Hao Yu; dateIdentified: 2026; **Event:** samplingProtocol: beating; samplingEffort: 10 km by foot; eventDate: 6/6/2022; **Record Level:** language: en; basisOfRecord: PreservedSpecimen; **Material Entity:** preparations: whole animal (ETOH)

#### Description

**Female (holotype)**. Living female with reddish-brown carapace and light brown legs, abdomen dorsally with a coffee-coloured median band and two yellow lateral stripes (Fig. 2A and B). Habitus in alcohol as in Fig. 6A–C. Total length 4.69. Carapace 1.51 long, 1.03 wide, primarily yellowish-white, with a wide median band, which is slightly narrower in the mid-section. Median band mainly brown, anteriorly with a V-shaped marking behind PME and PLE, almost reaching fovea; posteriorly bears a nearly triangular patch that extends to the posterior margin of the carapace. Cervical groove, fovea and radial furrow distinct. Clypeus 0.10 high. Mouthparts: chelicerae unmodified, coloured as carapace, with four teeth on promargin and three teeth on retromargin; endite length 0.38, width 0.28, depressed posteriorly, nearly parallel, with dense setae on inner margin; labium nearly triangular, length 0.10, width 0.24. Sternum 0.89 long, 0.69 wide, light yellow, smooth and without pattern. Eyes: AER (anterior eye row)

recurved, posterior eye row (PER) almost straight. Eye sizes and interdistances: AME (anterior median eyes) 0.08, ALE (anterior lateral eyes) 0.06, PME (posterior median eyes) 0.09, PLE (posterior lateral eyes) 0.09, distance between AMEs (AME–AME) 0.04, distance between PMEs (PME–PME) 0.09, distance between AME and ALE (AME–ALE) 0.09, distance between PME and PLE (PME–PLE) 0.10, distance between AME and PME (AME–PME) 0.13, ALE and PLE contiguous. Leg uniformly yellowish. Length of legs: I — (2.92, 0.35, —, —, —), II missing, III — (1.43, 0.26, —, —, —), IV — (2.31, 0.34, —, —, —). Opisthosoma 3.32 long, 1.27 wide, cylindrical, dorsally with a broad, light brown, longitudinal median band, extending almost entire length, posteriorly black; laterally with two white longitudinal stripes, which are nearly as long as median band; ventrally mainly flesh-coloured, with two black spots on each side; all spinnerets light brown, with a black spot present posteriorly.

Epigyne (Fig. 7A–G): Epigynal field ca. 1.3 × wider than long, anterior and lateral margin not rebordered; anterior wall mid-ventrally with a tuft of thick spines; the arrangement of the various parts of the vulva are distinctly visible through the transparent anterior wall after dissection. Atrium (A) located posteriorly, large, ca. 3/5 of epigyne width; anterior margin distinctly delimited recurved, posteriorly not rebordered. Copulatory openings (CO) indistinct, located at anterolateral atrial borders. Scape (Sc) protruding, hyaline, proximally sligntly narrowed while distally widened, more or less fan-shaped. Spiral ducts (SD) with ca. 2 turns; turning points (TP) and spermathecae (S) present at apex anteriorly. Spermathecae (S) present anteriorly at the apex of spiral ducts, digitiform, pointing away from each other. Turning points (TP) small, papilliform, located ventroposteriorly to spermathecae. Fertilisation ducts acicular, membranous, located on dorsal-basal portion of spiral ducts.

#### Diagnosis

The new species is similar to *L. longiabdominis* Irfan, Zhang & Peng, 2025 (the other only *Lenis* species: Irfan et al. (2025): 92, figs. 123A–D, 124D and E) in the general appearance of the atrium and endogyne. From *L. longiabdominis*, the female of the new species can be easily distinguished by the shape of the spermathecae and the different number of turns of spiral ducts: (1) spermathecae digitiform, barely curved (Fig. 7B, D, E and G) (vs. crescent-shaped, distinctly curved; Irfan et al. (2025): 92, figs. 123B and D); (2) spiral ducts (SD) with ca. 2 turns (Fig. 7B, D, E and G) (vs. ca. 2.5 turns; Irfan et al. (2025): 92, figs. 123B and D). In addition, the two species can by separated by their habitus: abdominal median band uniformly coloured in *L. guiyang* sp. nov. (Fig. 6A), but with three pairs of patches located at lateral part of median band in *L. longiabdominis* (Irfan et al. (2025): 92, fig. 124D).

#### Etymology

The species name is derived from the name of the type locality; noun in apposition.

#### Distribution

Known only from the type locality, Guiyang City, Guizhou Province, China (Fig. 1B).

### Bifurcia ramosa

(Li & Zhu, 1987)

C1B7C1E5-5991-5559-B5A1-AD3DDF3A64B8

#### Materials

- **Type status:** Other material. **Occurrence:** recordedBy: Qianle Lu; individualCount: 3; sex: 1 male, 2 females; lifeStage: adult; occurrenceID: 0AF1DF2D-21A3-5F17-9BC9-E6185FC796AA; **Taxon:** scientificName: *Bifurcia ramosa*; order: Araneae; family: Linyphiidae; genus: *Bifurcia*; specificEpithet: *ramosa*; scientificNameAuthorship: Li & Zhu; taxonomicStatus: accepted; **Location:** continent: Asia; country: China; countryCode: CHN; stateProvince: Guizhou; county: Xiuwen County; municipality: Guiyang; locality: Liutong Town, Duobing Cave; verbatimElevation: 1026 m; verbatimCoordinates: 27.10°N, 106.50°E; decimalLatitude: 27.10; decimalLongitude: 106.50; georeferenceProtocol: label; **Identification:** identifiedBy: Hao Yu; dateIdentified: 2022; **Event:** samplingProtocol: searching; samplingEffort: 10 km by foot; eventDate: 5/6/2022; **Record Level:** language: en; basisOfRecord: PreservedSpecimen

#### Description

See Saaristo et al. (2006). Live specimens as in Fig. 2C and D.

#### Distribution

China (Guizhou, Hubei, Hunan; distribution record in Guiyang as in Fig. 1B).

### Gnathonarium dentatum

(Wider, 1834)

03AA9CF5-B92D-57F0-B1BF-5D206BA630BF

#### Materials

- **Type status:** Other material. **Occurrence:** recordedBy: Qianle Lu; individualCount: 1; sex: female; lifeStage: adult; occurrenceID: 39C78CA8-0068-5631-9AA7-50F0570A9E02; **Taxon:** scientificName: *Gnathonarium dentatum*; order: Araneae; family: Linyphiidae; genus: *Gnathonarium*; specificEpithet: *dentatum*; scientificNameAuthorship: Wider; taxonomicStatus: accepted; **Location:** continent: Asia; country: China; countryCode: CHN; stateProvince: Guizhou; municipality: Guiyang; locality: Qian Tao Bou Yi and Miao Ethnic Township, Laobanghe; verbatimElevation: 1022 m; verbatimCoordinates: 26.34°N, 106.73°E; decimalLatitude: 26.34; decimalLongitude: 106.73; georeferenceProtocol: label; **Identification:** identifiedBy: Hao Yu; dateIdentified: 2022; **Event:** samplingProtocol: beating; samplingEffort: 10 km by foot; eventDate: 18/5/2022; **Record Level:** language: en; basisOfRecord: PreservedSpecimen
- **Type status:** Other material. **Occurrence:** recordedBy: Qianle Lu; individualCount: 1; sex: male; lifeStage: adult; occurrenceID: 17547FDB-EF2D-5AA6-968D-79AAFECF7370; **Taxon:** scientificName: *Gnathonarium dentatum*; order: Araneae; family: Linyphiidae; genus: *Gnathonarium*; specificEpithet: *dentatum*; scientificNameAuthorship: Wider; taxonomicStatus: accepted; **Location:** continent: Asia; country: China; countryCode: CHN; stateProvince: Guizhou; municipality: Guiyang; locality: Guizhou Province Botanical Garden; verbatimElevation: 1249 m; verbatimCoordinates: 26.63°N, 106.73°E; decimalLatitude: 26.63; decimalLongitude: 106.73; georeferenceProtocol: label; **Identification:** identifiedBy: Hao Yu; dateIdentified: 2022; **Event:** samplingProtocol: beating; samplingEffort: 10 km by foot; eventDate: 4/6/2022; **Record Level:** language: en; basisOfRecord: PreservedSpecimen
- **Type status:** Other material. **Occurrence:** recordedBy: Qianle Lu; individualCount: 2; sex: 1 male, 1 female; lifeStage: adult; occurrenceID: 975ECB29-6B81-5298-92F7-E337DB007964; **Taxon:** scientificName: *Gnathonarium dentatum*; order: Araneae; family: Linyphiidae; genus: *Gnathonarium*; specificEpithet: *dentatum*; scientificNameAuthorship: Wider; taxonomicStatus: accepted; **Location:** continent: Asia; country: China; countryCode: CHN; stateProvince: Guizhou; municipality: Guiyang; locality: Xinpu Buyi Ethnic Township, Xiangzhigou Scenic Spot, Guodijing Canyon; verbatimElevation: 1059 m; verbatimCoordinates: 26.79°N, 106.91°E; decimalLatitude: 26.79; decimalLongitude: 106.91; georeferenceProtocol: label; **Identification:** identifiedBy: Hao Yu; dateIdentified: 2022; **Event:** samplingProtocol: beating; samplingEffort: 10 km by foot; eventDate: 31/5/2022; **Record Level:** language: en; basisOfRecord: PreservedSpecimen

#### Description

See Tu and Li (2004). Live specimen as in Fig. 2E.

#### Distribution

China (Guizhou, Anhui, Beijing, Fujian, Gansu, Guangdong, Hebei, Henan, Hubei, Hunan, Inner Mongolia, Jiangsu, Jiangxi, Jilin, Ningxia, Qinghai, Shaanxi, Shandong, Shanxi, Sichuan, Xizang, Yunnan, Zhejiang; distribution records in Guiyang as in Fig. 1B), Europe, North Africa, Turkey, Israel, Caucasus, Russia (Europe to Far East), Kazakhstan, Iran, Central Asia, Korea, Japan.

### Gnathonarium taczanowskii

(O. Pickard-Cambridge, 1873)

C3F02767-B82B-5A66-BD00-5674F87F1E3A

#### Materials

- **Type status:** Other material. **Occurrence:** recordedBy: Qianle Lu; individualCount: 2; sex: 2 females; lifeStage: adult; occurrenceID: B8DE4E5C-63E7-5652-9524-4536276B5A2B; **Taxon:** scientificName: *Gnathonarium taczanowskii*; order: Araneae; family: Linyphiidae; genus: *Gnathonarium*; specificEpithet: *taczanowskii*; scientificNameAuthorship: O. Pickard-Cambridge; taxonomicStatus: accepted; **Location:** continent: Asia; country: China; countryCode: CHN; stateProvince: Guizhou; municipality: Guiyang; locality: Xinpu Buyi Ethnic Township, Xiangzhigou Scenic Spot, Guodijing Canyon; verbatimElevation: 1059 m; verbatimCoordinates: 26.79°N, 106.91°E; decimalLatitude: 26.79; decimalLongitude: 106.91; georeferenceProtocol: label; **Identification:** identifiedBy: Hao Yu; dateIdentified: 2022; **Event:** samplingProtocol: beating; samplingEffort: 10 km by foot; eventDate: 31/5/2022; **Record Level:** language: en; basisOfRecord: PreservedSpecimen
- **Type status:** Other material. **Occurrence:** recordedBy: Qianle Lu; individualCount: 1; sex: male; lifeStage: adult; occurrenceID: 314CA85C-D870-5B3A-AA38-8DA90743EC3D; **Taxon:** scientificName: *Gnathonarium taczanowskii*; order: Araneae; family: Linyphiidae; genus: *Gnathonarium*; specificEpithet: *taczanowskii*; scientificNameAuthorship: O. Pickard-Cambridge; taxonomicStatus: accepted; **Location:** continent: Asia; country: China; countryCode: CHN; stateProvince: Guizhou; municipality: Guiyang; locality: Gaopuo Miao Ethnic Township, Diba; verbatimElevation: 1216 m; verbatimCoordinates: 26.26°N, 106.85°E; decimalLatitude: 26.26; decimalLongitude: 106.85; georeferenceProtocol: label; **Identification:** identifiedBy: Hao Yu; dateIdentified: 2022; **Event:** samplingProtocol: beating; samplingEffort: 10 km by foot; eventDate: 21/5/2022; **Record Level:** language: en; basisOfRecord: PreservedSpecimen

#### Description

See Tu and Li (2004) (described under the name of *Gnathonarium cambridgei*).

#### Distribution

China (Guizhou, Gansu, Hebei, Henan, Hubei, Hunan, Jilin, Qinghai, Shaanxi, Shandong, Shanxi, Yunnan; distribution records in Guiyang as in Fig. 1B), Russia (Urals to the Far East), Kazakhstan, Mongolia, Alaska, Canada.

### Hylyphantes graminicola

(Sundevall, 1830)

02182DF4-7ECF-53B8-95C4-B3B38C9FF00D

#### Materials

- **Type status:** Other material. **Occurrence:** recordedBy: Qianle Lu; individualCount: 2; sex: 1 male, 1 female; lifeStage: adult; occurrenceID: 9F8F4169-5F20-51CA-9C78-574ED1CE2213; **Taxon:** scientificName: *Hylyphantes graminicola*; order: Araneae; family: Linyphiidae; genus: *Hylyphantes*; specificEpithet: *graminicola*; scientificNameAuthorship: Sundevall; taxonomicStatus: accepted; **Location:** continent: Asia; country: China; countryCode: CHN; stateProvince: Guizhou; municipality: Guiyang; locality: Poly Hot Spring Pack; verbatimElevation: 1138 m; verbatimCoordinates: 26.63°N, 106.75°E; decimalLatitude: 26.63; decimalLongitude: 106.75; georeferenceProtocol: label; **Identification:** identifiedBy: Hao Yu; dateIdentified: 2016; **Event:** samplingProtocol: beating; samplingEffort: 10 km by foot; eventDate: 16/5/2016; **Record Level:** language: en; basisOfRecord: PreservedSpecimen
- **Type status:** Other material. **Occurrence:** recordedBy: Qianle Lu; individualCount: 3; sex: 1 male, 2 females; lifeStage: adult; occurrenceID: 6A64B7ED-E91F-5C70-B2AB-B7DD351EAF39; **Taxon:** scientificName: *Hylyphantes graminicola*; order: Araneae; family: Linyphiidae; genus: *Hylyphantes*; specificEpithet: *graminicola*; scientificNameAuthorship: Sundevall; taxonomicStatus: accepted; **Location:** continent: Asia; country: China; countryCode: CHN; stateProvince: Guizhou; county: Kaiyang County; municipality: Guiyang; locality: Nanjiang Grand Canyon; verbatimElevation: 861 m; verbatimCoordinates: 26.94°N, 106.97°E; decimalLatitude: 26.94; decimalLongitude: 106.97; georeferenceProtocol: label; **Identification:** identifiedBy: Hao Yu; dateIdentified: 2022; **Event:** samplingProtocol: beating; samplingEffort: 10 km by foot; eventDate: 7/6/2022; **Record Level:** language: en; basisOfRecord: PreservedSpecimen
- **Type status:** Other material. **Occurrence:** recordedBy: Qianle Lu; individualCount: 1; sex: male; lifeStage: adult; occurrenceID: 4711C422-D84B-5618-A7FB-A5C53331F822; **Taxon:** scientificName: *Hylyphantes graminicola*; order: Araneae; family: Linyphiidae; genus: *Hylyphantes*; specificEpithet: *graminicola*; scientificNameAuthorship: Sundevall; taxonomicStatus: accepted; **Location:** continent: Asia; country: China; countryCode: CHN; stateProvince: Guizhou; municipality: Guiyang; locality: Dangwu Township, Xiaba Village; verbatimElevation: 1138 m; verbatimCoordinates: 26.38°N, 106.60°E; decimalLatitude: 26.38; decimalLongitude: 106.6; georeferenceProtocol: label; **Identification:** identifiedBy: Hao Yu; dateIdentified: 2022; **Event:** samplingProtocol: beating; samplingEffort: 10 km by foot; eventDate: 19/5/2022; **Record Level:** language: en; basisOfRecord: PreservedSpecimen

#### Description

See Tu and Li (2003). Live specimens as in Fig. 3A and B.

#### Distribution

China (Guizhou, Anhui, Fujian, Guangdong, Guangxi, Hebei, Henan, Hong Kong, Hubei, Hunan, Jiangsu, Jiangxi, Jilin, Liaoning, Ningxia, Qinghai, Shaanxi, Shandong, Shanghai, Shanxi, Sichuan, Taiwan, Xinjiang, Yunnan, Zhejiang; distribution records in Guiyang as in Fig. 1B), Europe, Russia (Europe to Far East), Nepal, Korea, Japan, Myanmar, Thailand, Laos, Vietnam.

### Ketambea nigripectoris

(Oi, 1960)

0268EAEF-0DA0-5D83-99A7-2AD1A656B8E5

#### Materials

- **Type status:** Other material. **Occurrence:** recordedBy: Qianle Lu; individualCount: 9; sex: 4 males, 5 females; lifeStage: adult; occurrenceID: ACE23E40-E9DE-5EAD-88B3-4D176BC1B2E1; **Taxon:** scientificName: *Ketambea nigripectoris*; order: Araneae; family: Linyphiidae; genus: *Ketambea*; specificEpithet: *nigripectoris*; scientificNameAuthorship: Oi; taxonomicStatus: accepted; **Location:** continent: Asia; country: China; countryCode: CHN; stateProvince: Guizhou; municipality: Guiyang; locality: Gaopuo Miao Ethnic Township, Soupo; verbatimElevation: 1358 m; verbatimCoordinates: 26.26°N, 106.83°E; decimalLatitude: 26.26; decimalLongitude: 106.83; georeferenceProtocol: label; **Identification:** identifiedBy: Hao Yu; dateIdentified: 2022; **Event:** samplingProtocol: beating; samplingEffort: 10 km by foot; eventDate: 21/5/2022; **Record Level:** language: en; basisOfRecord: PreservedSpecimen
- **Type status:** Other material. **Occurrence:** recordedBy: Qianle Lu; individualCount: 1; sex: female; lifeStage: adult; occurrenceID: 5AF3B5AD-D971-5114-B121-C1CC3DD2B7E9; **Taxon:** scientificName: *Ketambea nigripectoris*; order: Araneae; family: Linyphiidae; genus: *Ketambea*; specificEpithet: *nigripectoris*; scientificNameAuthorship: Oi; taxonomicStatus: accepted; **Location:** continent: Asia; country: China; countryCode: CHN; stateProvince: Guizhou; county: Kaiyang County; municipality: Guiyang; locality: Longgang Town, Pingshan Villagge, Zijang Rift Scenic Spot; verbatimElevation: 1124 m; verbatimCoordinates: 26.90°N, 107.03°E; decimalLatitude: 26.90; decimalLongitude: 107.03; georeferenceProtocol: label; **Identification:** identifiedBy: Hao Yu; dateIdentified: 2022; **Event:** samplingProtocol: beating; samplingEffort: 10 km by foot; eventDate: 8/6/2022; **Record Level:** language: en; basisOfRecord: PreservedSpecimen

#### Description

See Li et al. 2018. Live specimens as in Fig. 3C and D.

#### Distribution

China (Guizhou, Anhui, Chongqing, Fujian, Guangdong, Guangxi, Hebei, Hubei, Hunan, Jiangxi, Jilin, Sichuan, Zhejiang; distribution records in Guiyang as in Fig. 1B), Russia (Far East), Korea, Japan.

### Neriene aquilirostralis

Chen & Zhu, 1989

D41DC95F-8B1F-5657-912B-D1677AE1FB5B

#### Materials

- **Type status:** Other material. **Occurrence:** recordedBy: Qianle Lu; individualCount: 1; sex: female; lifeStage: adult; occurrenceID: A8F45AC6-828A-5379-B311-74EA6074F481; **Taxon:** scientificName: *Neriene aquilirostralis*; order: Araneae; family: Linyphiidae; genus: *Neriene*; specificEpithet: *aquilirostralis*; scientificNameAuthorship: Chen & Zhu; taxonomicStatus: accepted; **Location:** continent: Asia; country: China; countryCode: CHN; stateProvince: Guizhou; county: Xifeng County; municipality: Guiyang; locality: Mt. Xiwang, Fengchi Temple; verbatimElevation: 1437 m; verbatimCoordinates: 27.13°N, 106.64°E; decimalLatitude: 27.13; decimalLongitude: 106.64; georeferenceProtocol: label; **Identification:** identifiedBy: Hao Yu; dateIdentified: 2022; **Event:** samplingProtocol: beating; samplingEffort: 10 km by foot; eventDate: 6/6/2022; **Record Level:** language: en; basisOfRecord: PreservedSpecimen
- **Type status:** Other material. **Occurrence:** recordedBy: Qianle Lu; individualCount: 1; sex: female; lifeStage: adult; occurrenceID: 34CA32F0-F5DC-5D7E-A124-267B557FDDF6; **Taxon:** scientificName: *Neriene aquilirostralis*; order: Araneae; family: Linyphiidae; genus: *Neriene*; specificEpithet: *aquilirostralis*; scientificNameAuthorship: Chen & Zhu; taxonomicStatus: accepted; **Location:** continent: Asia; country: China; countryCode: CHN; stateProvince: Guizhou; municipality: Guiyang; locality: Guizhou Province Botanical Garden; verbatimElevation: 1249 m; verbatimCoordinates: 26.63°N, 106.73°E; decimalLatitude: 26.63; decimalLongitude: 106.73; georeferenceProtocol: label; **Identification:** identifiedBy: Hao Yu; dateIdentified: 2022; **Event:** samplingProtocol: beating; samplingEffort: 10 km by foot; eventDate: 4/6/2022; **Record Level:** language: en; basisOfRecord: PreservedSpecimen
- **Type status:** Other material. **Occurrence:** recordedBy: Qianle Lu; individualCount: 1; sex: male; lifeStage: adult; occurrenceID: EE78DAA1-635B-5F00-AAB4-F984E3EA2887; **Taxon:** scientificName: *Neriene aquilirostralis*; order: Araneae; family: Linyphiidae; genus: *Neriene*; specificEpithet: *aquilirostralis*; scientificNameAuthorship: Chen & Zhu; taxonomicStatus: accepted; **Location:** continent: Asia; country: China; countryCode: CHN; stateProvince: Guizhou; county: Xiuwen County; municipality: Guiyang; locality: Liutong Town, Duobing Cave; verbatimElevation: 1026 m; verbatimCoordinates: 27.10°N, 106.50°E; decimalLatitude: 27.10 ; decimalLongitude: 106.50 ; georeferenceProtocol: label; **Identification:** identifiedBy: Hao Yu; dateIdentified: 2023; **Event:** samplingProtocol: searching; samplingEffort: 11 km by foot; eventDate: 5/6/2022; **Record Level:** language: en; basisOfRecord: PreservedSpecimen

#### Description

See Li et al. (2018). Live specimens as in Fig. 3E and F.

#### Distribution

China (Guizhou, Chongqing, Hebei, Hubei, Shaanxi; distribution records in Guiyang as in Fig. 1B), Korea.

### Neriene cavaleriei

(Schenkel, 1963)

285B2EE3-694C-538B-B3DB-467C4BA62F91

#### Materials

- **Type status:** Other material. **Occurrence:** recordedBy: Qianle Lu; individualCount: 2; sex: 2 females; lifeStage: adult; occurrenceID: F49E080D-B519-56EA-860F-9828AD3245DD; **Taxon:** scientificName: *Neriene cavaleriei*; order: Araneae; family: Linyphiidae; genus: *Neriene*; specificEpithet: *cavaleriei*; scientificNameAuthorship: Schenkel; taxonomicStatus: accepted; **Location:** continent: Asia; country: China; countryCode: CHN; stateProvince: Guizhou; municipality: Guiyang; locality: Gaopuo Miao Ethnic Township, Sanchahe; verbatimElevation: 1162 m; verbatimCoordinates: 26.27°N, 106.80°E; decimalLatitude: 26.27; decimalLongitude: 106.80 ; georeferenceProtocol: label; **Identification:** identifiedBy: Hao Yu; dateIdentified: 2022; **Event:** samplingProtocol: beating; samplingEffort: 10 km by foot; eventDate: 20/5/2022; **Record Level:** language: en; basisOfRecord: PreservedSpecimen
- **Type status:** Other material. **Occurrence:** recordedBy: Hao Yu; individualCount: 1; sex: male; lifeStage: adult; occurrenceID: 638DFB4E-BD19-5A66-9BDF-F14B48C0DC5B; **Taxon:** scientificName: *Neriene cavaleriei*; order: Araneae; family: Linyphiidae; genus: *Neriene*; specificEpithet: *cavaleriei*; scientificNameAuthorship: Schenkel; taxonomicStatus: accepted; **Location:** continent: Asia; country: China; countryCode: CHN; stateProvince: Guizhou; municipality: Guiyang; locality: Xinzhuang Village; verbatimElevation: 1038 m; verbatimCoordinates: 26.64°N, 106.80°E; decimalLatitude: 26.64; decimalLongitude: 106.80; georeferenceProtocol: label; **Identification:** identifiedBy: Hao Yu; dateIdentified: 2016; **Event:** samplingProtocol: beating; samplingEffort: 10 km by foot; eventDate: 17/5/2016; **Record Level:** language: en; basisOfRecord: PreservedSpecimen

#### Description

See Tu and Li (2006). Live specimens as in Fig. 4A and B.

#### Distribution

China (Guizhou, Guangdong, Guangxi, Fujian, Zhejiang, Hunan, Hubei, Sichuan, Chongqing, Gansu, Hebei; distribution records in Guiyang as in Fig. 1B), Vietnam.

### Neriene japonica

(Oi, 1960)

B2925D4B-6EB8-5AC7-ABA2-E975762BB744

#### Materials

- **Type status:** Other material. **Occurrence:** recordedBy: Qianle Lu; individualCount: 2; sex: 1 male, 1 female; lifeStage: adult; occurrenceID: AED3E203-E8D6-53BC-9EC4-CF6843A6DECF; **Taxon:** scientificName: *Neriene japonica*; order: Araneae; family: Linyphiidae; genus: *Neriene*; specificEpithet: *japonica*; scientificNameAuthorship: Oi; taxonomicStatus: accepted; **Location:** continent: Asia; country: China; countryCode: CHN; stateProvince: Guizhou; municipality: Guiyang; locality: Gaopo Miao Ethnic Township, Soupo; verbatimElevation: 1358 m; verbatimCoordinates: 26.26°N, 106.83°E; decimalLatitude: 26.26; decimalLongitude: 106.83; georeferenceProtocol: label; **Identification:** identifiedBy: Hao Yu; dateIdentified: 2022; **Event:** samplingProtocol: beating; samplingEffort: 10 km by foot; eventDate: 21/5/2022; **Record Level:** language: en; basisOfRecord: PreservedSpecimen
- **Type status:** Other material. **Occurrence:** recordedBy: Hao Yu; individualCount: 1; sex: male; lifeStage: adult; occurrenceID: 6B204A6B-D51C-5B0E-92B0-90B9F48BDBA6; **Taxon:** scientificName: *Neriene japonica*; order: Araneae; family: Linyphiidae; genus: *Neriene*; specificEpithet: *japonica*; scientificNameAuthorship: Oi; taxonomicStatus: accepted; **Location:** continent: Asia; country: China; countryCode: CHN; stateProvince: Guizhou; municipality: Guiyang; locality: Pianpo Buyi Ethnic Township; verbatimElevation: 1308 m; verbatimCoordinates: 26.66°N, 106.93°E; decimalLatitude: 26.66; decimalLongitude: 106.93; georeferenceProtocol: label; **Identification:** identifiedBy: Hao Yu; dateIdentified: 2021; **Event:** samplingProtocol: beating; samplingEffort: 10 km by foot; eventDate: 8/8/2021; **Record Level:** language: en; basisOfRecord: PreservedSpecimen

#### Description

See Helsdingen (1969). Live specimens as in Fig. 4C and D.

#### Distribution

China (Guizhou, Anhui, Chongqing, Guangdong, Guangxi, Hebei, Heilongjiang, Henan, Hubei, Hunan, Jiangsu, Jiangxi, Jilin, Liaoning, Shaanxi, Shanxi, Sichuan, Zhejiang; distribution records in Guiyang as in Fig. 1B), Russia (Far East), Korea, Japan.

### Neriene longipedella

(Bösenberg & Strand, 1906)

27257CF6-91EA-5AAB-8F49-C2B9DCF0EB3A

#### Materials

- **Type status:** Other material. **Occurrence:** recordedBy: Hao Yu; individualCount: 3; sex: 2 males, 1 female; lifeStage: adult; occurrenceID: 9F1003FD-0017-56AD-A032-50BE63FF6C59; **Taxon:** scientificName: *Neriene longipedella*; order: Araneae; family: Linyphiidae; genus: *Neriene*; specificEpithet: *longipedella*; scientificNameAuthorship: Bösenberg & Strand; taxonomicStatus: accepted; **Location:** continent: Asia; country: China; countryCode: CHN; stateProvince: Guizhou; municipality: Guiyang; locality: Panlong Mountain Forest Park; verbatimElevation: 1215 m; verbatimCoordinates: 26.74°N, 106.86°E; decimalLatitude: 26.74; decimalLongitude: 106.86; georeferenceProtocol: label; **Identification:** identifiedBy: Hao Yu; dateIdentified: 2021; **Event:** samplingProtocol: beating; samplingEffort: 10 km by foot; eventDate: 7/8/2021; **Record Level:** language: en; basisOfRecord: PreservedSpecimen
- **Type status:** Other material. **Occurrence:** recordedBy: Qianle Lu; individualCount: 8; sex: 1 male, 7 females; lifeStage: adult; occurrenceID: CC9497EF-4549-582F-AAB5-15699D1E09BC; **Taxon:** scientificName: *Neriene longipedella*; order: Araneae; family: Linyphiidae; genus: *Neriene*; specificEpithet: *longipedella*; scientificNameAuthorship: Bösenberg & Strand; taxonomicStatus: accepted; **Location:** continent: Asia; country: China; countryCode: CHN; stateProvince: Guizhou; municipality: Guiyang; locality: Poly Hot Spring Park; verbatimElevation: 1310 m; verbatimCoordinates: 26.63°N, 106.75°E; decimalLatitude: 26.63; decimalLongitude: 106.75; georeferenceProtocol: label; **Identification:** identifiedBy: Hao Yu; dateIdentified: 2016; **Event:** samplingProtocol: beating; samplingEffort: 10 km by foot; eventDate: 16/5/2016; **Record Level:** language: en; basisOfRecord: PreservedSpecimen

#### Description

See Helsdingen (1969). Live specimens as in Fig. 4E and F.

#### Distribution

China (Guizhou, Anhui, Chongqing, Gansu, Hebei, Heilongjiang, Hubei, Hunan, Jilin, Shaanxi, Shanxi, Sichuan, Zhejiang; distribution records in Guiyang as in Fig. 1B), Russia (Far East), Korea, Japan.

### Neriene nitens

Zhu & Chen, 1991

BF853DAF-F92F-5AE3-A0B7-6EE1EAD05E46

#### Materials

- **Type status:** Other material. **Occurrence:** recordedBy: Hao Yu; individualCount: 2; sex: 2 males; lifeStage: adult; occurrenceID: 841DDF14-DA37-566A-807F-DDBDF133A7A2; **Taxon:** scientificName: *Neriene nitens*; order: Araneae; family: Linyphiidae; genus: *Neriene*; specificEpithet: *nitens*; scientificNameAuthorship: Zhu & Chen; taxonomicStatus: accepted; **Location:** continent: Asia; country: China; countryCode: CHN; stateProvince: Guizhou; municipality: Guiyang; locality: Panlong Mountain Forest Park; verbatimElevation: 1215 m; verbatimCoordinates: 26.74°N, 106.86°E; decimalLatitude: 26.74; decimalLongitude: 106.86; georeferenceProtocol: label; **Identification:** identifiedBy: Hao Yu; dateIdentified: 2021; **Event:** samplingProtocol: beating; samplingEffort: 10 km by foot; eventDate: 7/8/2021; **Record Level:** language: en; basisOfRecord: PreservedSpecimen
- **Type status:** Other material. **Occurrence:** recordedBy: Hao Yu; individualCount: 1; sex: female; lifeStage: adult; occurrenceID: 48C78387-56D9-579D-A213-E82BB4A8D124; **Taxon:** scientificName: *Neriene nitens*; order: Araneae; family: Linyphiidae; genus: *Neriene*; specificEpithet: *nitens*; scientificNameAuthorship: Zhu & Chen; taxonomicStatus: accepted; **Location:** continent: Asia; country: China; countryCode: CHN; stateProvince: Guizhou; municipality: Guiyang; locality: Qingrengu Scenic Spot ; verbatimElevation: 1088 m; verbatimCoordinates: 26.61°N, 106.80°E; decimalLatitude: 26.61; decimalLongitude: 106.80; georeferenceProtocol: label; **Identification:** identifiedBy: Hao Yu; dateIdentified: 2023; **Event:** samplingProtocol: beating; samplingEffort: 10 km by foot; eventDate: 27/6/2023; **Record Level:** language: en; basisOfRecord: PreservedSpecimen
- **Type status:** Other material. **Occurrence:** recordedBy: Hao Yu; individualCount: 27; sex: 18 males, 9 females; lifeStage: adult; occurrenceID: CD5A25C7-2DA5-5FFC-8663-171162B5C7EE; **Taxon:** scientificName: *Neriene nitens*; order: Araneae; family: Linyphiidae; genus: *Neriene*; specificEpithet: *nitens*; scientificNameAuthorship: Zhu & Chen; taxonomicStatus: accepted; **Location:** continent: Asia; country: China; countryCode: CHN; stateProvince: Guizhou; municipality: Guiyang; locality: Xinpu Buyi Ethnic Township, Xiangzhigou Scenic Spot, Guodijing Canyon; verbatimElevation: 1059 m; verbatimCoordinates: 26.79°N, 106.91°E; decimalLatitude: 26.79; decimalLongitude: 106.91; georeferenceProtocol: label; **Identification:** identifiedBy: Hao Yu; dateIdentified: 2023; **Event:** samplingProtocol: beating; samplingEffort: 10 km by foot; eventDate: 28/6/2023; **Record Level:** language: en; basisOfRecord: PreservedSpecimen

#### Description

See Li et al. (2018).

#### Distribution

China (Guizhou, Anhui, Fujian, Hubei, Hunan, Sichuan, Zhejiang; distribution records in Guiyang as in Fig. 1B).

### Neriene oidedicata

van Helsdingen, 1969

01CCB7B2-F099-5489-9904-7E6697047DF9

#### Materials

- **Type status:** Other material. **Occurrence:** recordedBy: Hao Yu; individualCount: 2; sex: 2 females; lifeStage: adult; occurrenceID: 276C0B8E-7D03-5B9D-83B5-42249D18A620; **Taxon:** scientificName: *Neriene oidedicata*; order: Araneae; family: Linyphiidae; genus: *Neriene*; specificEpithet: *oidedicata*; scientificNameAuthorship: van Helsdingen; taxonomicStatus: accepted; **Location:** continent: Asia; country: China; countryCode: CHN; stateProvince: Guizhou; municipality: Guiyang; locality: Guiyang Forest Park; verbatimElevation: 1165 m; verbatimCoordinates: 26.55°N, 106.75°E; decimalLatitude: 26.55; decimalLongitude: 106.75; georeferenceProtocol: label; **Identification:** identifiedBy: Hao Yu; dateIdentified: 2023; **Event:** samplingProtocol: beating; samplingEffort: 10 km by foot; eventDate: 10/8/2021; **Record Level:** language: en; basisOfRecord: PreservedSpecimen
- **Type status:** Other material. **Occurrence:** recordedBy: Hao Yu; individualCount: 4; sex: 1 male, 3 females; lifeStage: adult; occurrenceID: 15401191-0E0F-5FB4-A52F-494D0D18A73A; **Taxon:** scientificName: *Neriene oidedicata*; order: Araneae; family: Linyphiidae; genus: *Neriene*; specificEpithet: *oidedicata*; scientificNameAuthorship: van Helsdingen; taxonomicStatus: accepted; **Location:** continent: Asia; country: China; countryCode: CHN; stateProvince: Guizhou; municipality: Guiyang; locality: Pianpo Buyi Ethnic Township; verbatimElevation: 1308 m; verbatimCoordinates: 26.66°N, 106.93°E; decimalLatitude: 26.66; decimalLongitude: 106.93; georeferenceProtocol: label; **Identification:** identifiedBy: Hao Yu; dateIdentified: 2024; **Event:** samplingProtocol: beating; samplingEffort: 10 km by foot; eventDate: 8/8/2021; **Record Level:** language: en; basisOfRecord: PreservedSpecimen

#### Description

See Helsdingen (1969). Live specimen as in Fig. 2F.

#### Distribution

China (Guizhou, Anhui, Chongqing, Heilongjiang, Henan, Hubei, Hunan, Jiangsu, Jilin, Shandong, Sichuan, Taiwan, Zhejiang; distribution records in Guiyang as in Fig. 1B), Nepal, Russia (Far East), Korea, Japan.

### Parameioneta bilobata

Li & Zhu, 1993

C97FA782-C508-5D8D-A41A-57892E974606

#### Materials

- **Type status:** Other material. **Occurrence:** recordedBy: Hao Yu; individualCount: 1; sex: female; lifeStage: adult; occurrenceID: A54173A1-933A-56F4-98C5-3C2A4A4D56CB; **Taxon:** scientificName: *Parameioneta bilobata*; order: Araneae; family: Linyphiidae; genus: *Parameioneta*; specificEpithet: *bilobata*; scientificNameAuthorship: Li & Zhu; taxonomicStatus: accepted; **Location:** continent: Asia; country: China; countryCode: CHN; stateProvince: Guizhou; municipality: Guiyang; locality: Xinpu Buyi Ethnic Township, Xiangzhigou Scenic Spot, Guodijing Canyon; verbatimElevation: 1059 m; verbatimCoordinates: 26.79°N, 106.91°E; decimalLatitude: 26.79; decimalLongitude: 106.91; georeferenceProtocol: label; **Identification:** identifiedBy: Hao Yu; dateIdentified: 2023; **Event:** samplingProtocol: beating; samplingEffort: 10 km by foot; eventDate: 28/6/2023; **Record Level:** language: en; basisOfRecord: PreservedSpecimen

#### Description

See Tu and Li (2006).

#### Distribution

China (Guizhou, Fujian, Hubei, Hunan, Yunnan; distribution record in Guiyang as in Fig. 1B), Vietnam.

### Prosoponoides sinense

(Chen, 1991)

4DEE42B4-1AAE-5D5C-A63E-3F4BEBB9C2D6

#### Materials

- **Type status:** Other material. **Occurrence:** recordedBy: Hao Yu; individualCount: 7; sex: 3 males, 4 females; lifeStage: adult; occurrenceID: 9637B73F-4FEB-5504-B62D-CA3BA173ABAA; **Taxon:** scientificName: *Prosoponoides sinense*; order: Araneae; family: Linyphiidae; genus: *Prosoponoides*; specificEpithet: *sinense*; scientificNameAuthorship: Chen; taxonomicStatus: accepted; **Location:** continent: Asia; country: China; countryCode: CHN; stateProvince: Guizhou; municipality: Guiyang; locality: Baihuahu Town, Mt. Jiulong; verbatimElevation: 1512 m; verbatimCoordinates: 26.58°N, 106.43°E; decimalLatitude: 26.58; decimalLongitude: 106.43; georeferenceProtocol: label; **Identification:** identifiedBy: Hao Yu; dateIdentified: 2022; **Event:** samplingProtocol: beating; samplingEffort: 10 km by foot; eventDate: 4/9/2021; **Record Level:** language: en; basisOfRecord: PreservedSpecimen
- **Type status:** Other material. **Occurrence:** recordedBy: Qianle Lu; individualCount: 1; sex: female; lifeStage: adult; occurrenceID: 6A8351FA-C849-5B49-B6B1-0F554FB727EC; **Taxon:** scientificName: *Prosoponoides sinense*; order: Araneae; family: Linyphiidae; genus: *Prosoponoides*; specificEpithet: *sinensis*; scientificNameAuthorship: Chen; taxonomicStatus: accepted; **Location:** continent: Asia; country: China; countryCode: CHN; stateProvince: Guizhou; municipality: Guiyang; locality: Xinpu Buyi Ethnic Township, Xiangzhigou Scenic Spot, Longjingwan Canyon; verbatimElevation: 1092 m; verbatimCoordinates: 26.78°N, 106.92°E; decimalLatitude: 26.78; decimalLongitude: 106.92; georeferenceProtocol: label; **Identification:** identifiedBy: Hao Yu; dateIdentified: 2022; **Event:** samplingProtocol: beating; samplingEffort: 10 km by foot; eventDate: 1/6/2022; **Record Level:** language: en; basisOfRecord: PreservedSpecimen
- **Type status:** Other material. **Occurrence:** recordedBy: Hao Yu; individualCount: 3; sex: 3 females; lifeStage: adult; occurrenceID: 76BA1AFF-EFBD-5144-A7C3-CA54FAA6A491; **Taxon:** scientificName: *Prosoponoides sinense*; order: Araneae; family: Linyphiidae; genus: *Prosoponoides*; specificEpithet: *sinense*; scientificNameAuthorship: Chen; taxonomicStatus: accepted; **Location:** continent: Asia; country: China; countryCode: CHN; stateProvince: Guizhou; municipality: Guiyang; locality: Xinzhuang Village; verbatimElevation: 1038 m; verbatimCoordinates: 26.64°N, 106.80°E; decimalLatitude: 26.64; decimalLongitude: 106.80 ; georeferenceProtocol: label; **Identification:** identifiedBy: Hao Yu; dateIdentified: 2015; **Event:** samplingProtocol: beating; samplingEffort: 10 km by foot; eventDate: 17/5/2015; **Record Level:** language: en; basisOfRecord: PreservedSpecimen

#### Description

See Tu and Li (2006). Live specimens as in Fig. 5A and B.

#### Distribution

China (Guizhou, Chongqing, Fujian, Hubei, Hunan, Yunnan, Zhejiang; distribution records in Guiyang as in Fig. 1B), Vietnam, Malaysia (Peninsula).

### Sinolinyphia henanensis

(Hu, Wang & Wang, 1991)

60285B43-908A-5EBB-B569-C5A4BF01AF38

#### Materials

- **Type status:** Other material. **Occurrence:** recordedBy: Qianle Lu; individualCount: 4 ; sex: 1male, 3 females; lifeStage: adult; occurrenceID: 45CB31B1-6CF9-5110-B63D-FAE578701968; **Taxon:** scientificName: *Sinolinyphia henanensis*; order: Araneae; family: Linyphiidae; genus: *Sinolinyphia*; specificEpithet: *henanensis*; scientificNameAuthorship: Hu, Wang & Wang; taxonomicStatus: accepted; **Location:** continent: Asia; country: China; countryCode: CHN; stateProvince: Guizhou; municipality: Guiyang; locality: Luchongguan Forest Park; verbatimElevation: 1310 m; verbatimCoordinates: 26.63°N, 106.70°E; decimalLatitude: 26.63; decimalLongitude: 106.70 ; georeferenceProtocol: label; **Identification:** identifiedBy: Hao Yu; dateIdentified: 2023; **Event:** samplingProtocol: beating; samplingEffort: 10 km by foot; eventDate: 4/6/2022; **Record Level:** language: en; basisOfRecord: PreservedSpecimen

#### Description

See Marusik and Omelko (2021). Live specimens as in Fig. 5C and D.

#### Distribution

China (Guizhou, Hebei, Henan, Hubei, Hunan, Liaoning, Shaanxi, Zhejiang; distribution record in Guiyang as in Fig. 1B), Russia (Far East).

### Syedra oii

Saito, 1983

B4EB59DA-0A03-5EFA-80ED-F020F0758E74

#### Materials

- **Type status:** Other material. **Occurrence:** recordedBy: Hao Yu; individualCount: 1; sex: female; lifeStage: adult; occurrenceID: 50E103AC-9575-5AAD-B359-EC861B044CA6; **Taxon:** scientificName: *Syedra oii*; order: Araneae; family: Linyphiidae; genus: *Syedra*; specificEpithet: *oii*; scientificNameAuthorship: Saito; taxonomicStatus: accepted; **Location:** continent: Asia; country: China; countryCode: CHN; stateProvince: Guizhou; municipality: Guiyang; locality: Guiyang Forest Park; verbatimElevation: 1165 m; verbatimCoordinates: 26.55°N, 106.75°E; decimalLatitude: 26.55; decimalLongitude: 106.75; georeferenceProtocol: label; **Identification:** identifiedBy: Hao Yu; dateIdentified: 2023; **Event:** samplingProtocol: beating; samplingEffort: 10 km by foot; eventDate: 26/6/2023; **Record Level:** language: en; basisOfRecord: PreservedSpecimen
- **Type status:** Other material. **Occurrence:** recordedBy: Hao Yu; individualCount: 1; sex: female; lifeStage: adult; occurrenceID: 513508C6-0D12-55B8-9D70-33186EB71A55; **Taxon:** scientificName: *Syedra oii*; order: Araneae; family: Linyphiidae; genus: *Syedra*; specificEpithet: *oii*; scientificNameAuthorship: Saito; taxonomicStatus: accepted; **Location:** continent: Asia; country: China; countryCode: CHN; stateProvince: Guizhou; municipality: Guiyang; locality: Yudongxia Scenic Spot; verbatimElevation: 996 m; verbatimCoordinates: 26.64°N, 106.83°E; decimalLatitude: 26.64; decimalLongitude: 106.83; georeferenceProtocol: label; **Identification:** identifiedBy: Hao Yu; dateIdentified: 2023; **Event:** samplingProtocol: beating; samplingEffort: 10 km by foot; eventDate: 27/6/2023; **Record Level:** language: en; basisOfRecord: PreservedSpecimen

#### Description

See Yoo et al. (2004).

#### Distribution

China (Guizhou, Hunan, Zhejiang; distribution records in Guiyang as in Fig. 1B), Korea, Japan.

### Ummeliata feminea

(Bösenberg & Strand, 1906)

F7C517D3-0AFD-5731-AF6F-928C39FD533B

#### Materials

- **Type status:** Other material. **Occurrence:** recordedBy: Qianle Lu; individualCount: 2; sex: 1 male, 1 female; lifeStage: adult; occurrenceID: 398B7054-8CB0-56D7-8133-BADB2B67CA9D; **Taxon:** scientificName: *Ummeliata feminea*; order: Araneae; family: Linyphiidae; genus: *Ummeliata*; specificEpithet: *feminea*; scientificNameAuthorship: Bösenberg & Strand; taxonomicStatus: accepted; **Location:** continent: Asia; country: China; countryCode: CHN; stateProvince: Guizhou; county: Kaiyang County; municipality: Guiyang; locality: Nanjiang Grand Canyon; verbatimElevation: 861 m; verbatimCoordinates: 26.94°N, 106.97°E; decimalLatitude: 26.94; decimalLongitude: 106.97; georeferenceProtocol: label; **Identification:** identifiedBy: Hao Yu; dateIdentified: 2022; **Event:** samplingProtocol: beating; samplingEffort: 10 km by foot; eventDate: 7/6/2022; **Record Level:** language: en; basisOfRecord: PreservedSpecimen
- **Type status:** Other material. **Occurrence:** recordedBy: Qianle Lu; individualCount: 1; sex: female; lifeStage: adult; occurrenceID: 8F44AA20-9F74-5205-9D24-FB13C3F7026E; **Taxon:** scientificName: *Ummeliata feminea*; order: Araneae; family: Linyphiidae; genus: *Ummeliata*; specificEpithet: *feminea*; scientificNameAuthorship: Bösenberg & Strand; taxonomicStatus: accepted; **Location:** continent: Asia; country: China; countryCode: CHN; stateProvince: Guizhou; municipality: Guiyang; locality: Dangwu Township, Xiaba Village; verbatimElevation: 1138 m; verbatimCoordinates: 26.38°N, 106.60°E; decimalLatitude: 26.38; decimalLongitude: 106.60 ; georeferenceProtocol: label; **Identification:** identifiedBy: Hao Yu; dateIdentified: 2022; **Event:** samplingProtocol: beating; samplingEffort: 10 km by foot; eventDate: 19/5/2022; **Record Level:** language: en; basisOfRecord: PreservedSpecimen
- **Type status:** Other material. **Occurrence:** recordedBy: Hao Yu; individualCount: 1; sex: male; lifeStage: adult; occurrenceID: 24D4ACCB-86D8-5343-A329-D6B838A90BF2; **Taxon:** scientificName: *Ummeliata feminea*; order: Araneae; family: Linyphiidae; genus: *Ummeliata*; specificEpithet: *feminea*; scientificNameAuthorship: Bösenberg & Strand; taxonomicStatus: accepted; **Location:** continent: Asia; country: China; countryCode: CHN; stateProvince: Guizhou; municipality: Guiyang; locality: Qingrengu Scenic Spot ; verbatimElevation: 1088 m; verbatimCoordinates: 26.61°N, 106.80°E; decimalLatitude: 26.61; decimalLongitude: 106.80 ; georeferenceProtocol: label; **Identification:** identifiedBy: Hao Yu; dateIdentified: 2023; **Event:** samplingProtocol: beating; samplingEffort: 10 km by foot; eventDate: 6/27/2023; **Record Level:** language: en; basisOfRecord: PreservedSpecimen
- **Type status:** Other material. **Occurrence:** recordedBy: Hao Yu; individualCount: 1; sex: female; lifeStage: adult; occurrenceID: B96FB569-66B2-5197-8AE6-6BA4A4E9CFA3; **Taxon:** scientificName: *Ummeliata feminea*; order: Araneae; family: Linyphiidae; genus: *Ummeliata*; specificEpithet: *feminea*; scientificNameAuthorship: Bösenberg & Strand; taxonomicStatus: accepted; **Location:** continent: Asia; country: China; countryCode: CHN; stateProvince: Guizhou; municipality: Guiyang; locality: Yudongxia Scenic Spot; verbatimElevation: 996 m; verbatimCoordinates: 26.64°N, 106.83°E; decimalLatitude: 26.64; decimalLongitude: 106.83; georeferenceProtocol: label; **Identification:** identifiedBy: Hao Yu; dateIdentified: 2023; **Event:** samplingProtocol: beating; samplingEffort: 10 km by foot; eventDate: 27/6/2023; **Record Level:** language: en; basisOfRecord: PreservedSpecimen

#### Description

See Oi (1960) (described under the name of *Oedothorax tokyoensis*). Live specimens as in Fig. 5E and F.

#### Distribution

China (Guizhou, Beijing, Fujian, Gansu, Hebei, Henan, Hubei, Jilin, Qinghai, Shaanxi, Shandong, Shanxi, Yunnan; distribution records in Guiyang as in Fig. 1B), Russia (Far East), Korea, Japan.

## Supplementary Material

XML Treatment for Lenis guiyang

XML Treatment for Bifurcia ramosa

XML Treatment for Gnathonarium dentatum

XML Treatment for Gnathonarium taczanowskii

XML Treatment for Hylyphantes graminicola

XML Treatment for Ketambea nigripectoris

XML Treatment for Neriene aquilirostralis

XML Treatment for Neriene cavaleriei

XML Treatment for Neriene japonica

XML Treatment for Neriene longipedella

XML Treatment for Neriene nitens

XML Treatment for Neriene oidedicata

XML Treatment for Parameioneta bilobata

XML Treatment for Prosoponoides sinense

XML Treatment for Sinolinyphia henanensis

XML Treatment for Syedra oii

XML Treatment for Ummeliata feminea

## Figures and Tables

**Figure 1.**
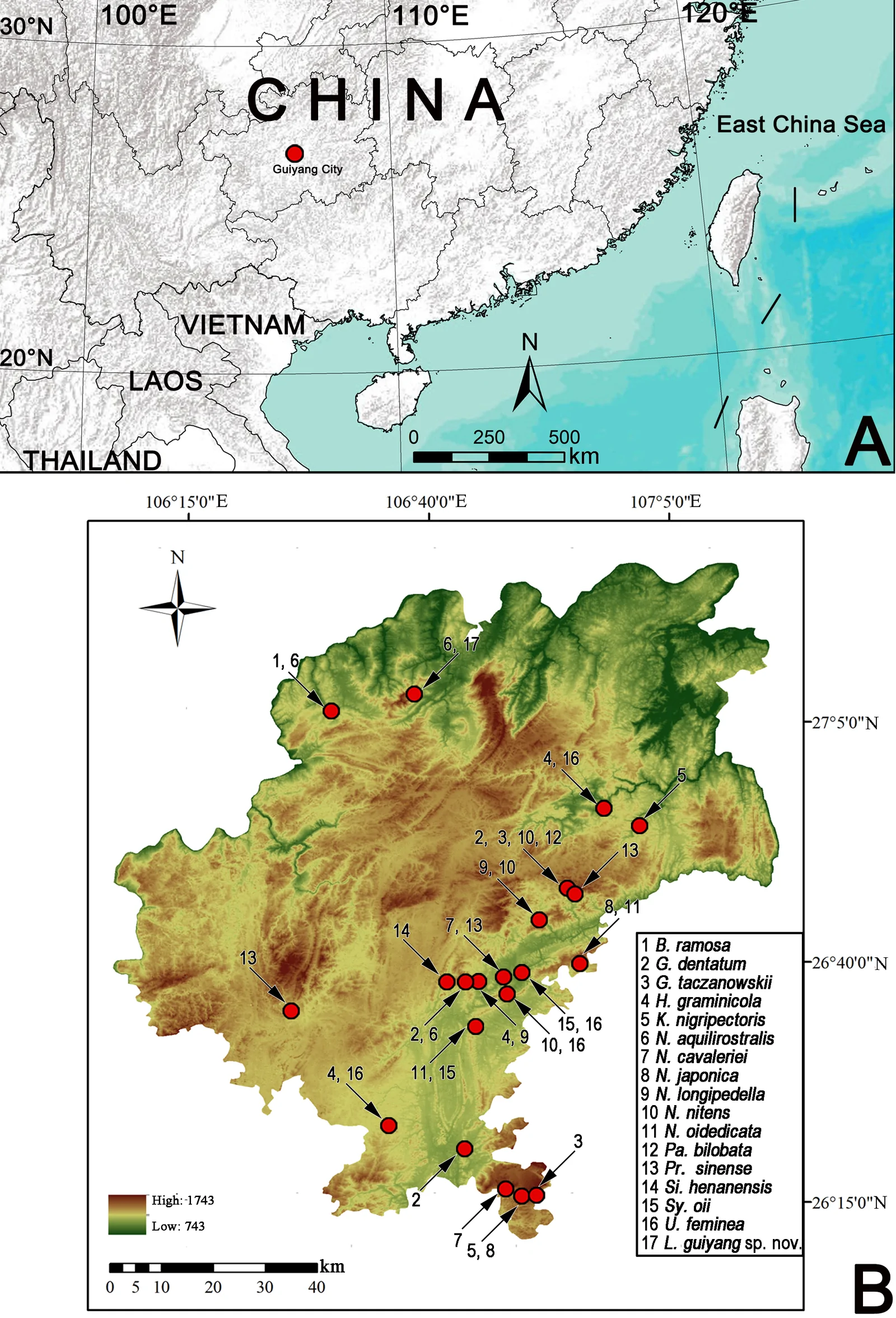
Locality of Guiyang City (**A**) and distribution records of the linyphiid species in Guiyang (**B**).

**Figure 2a.**
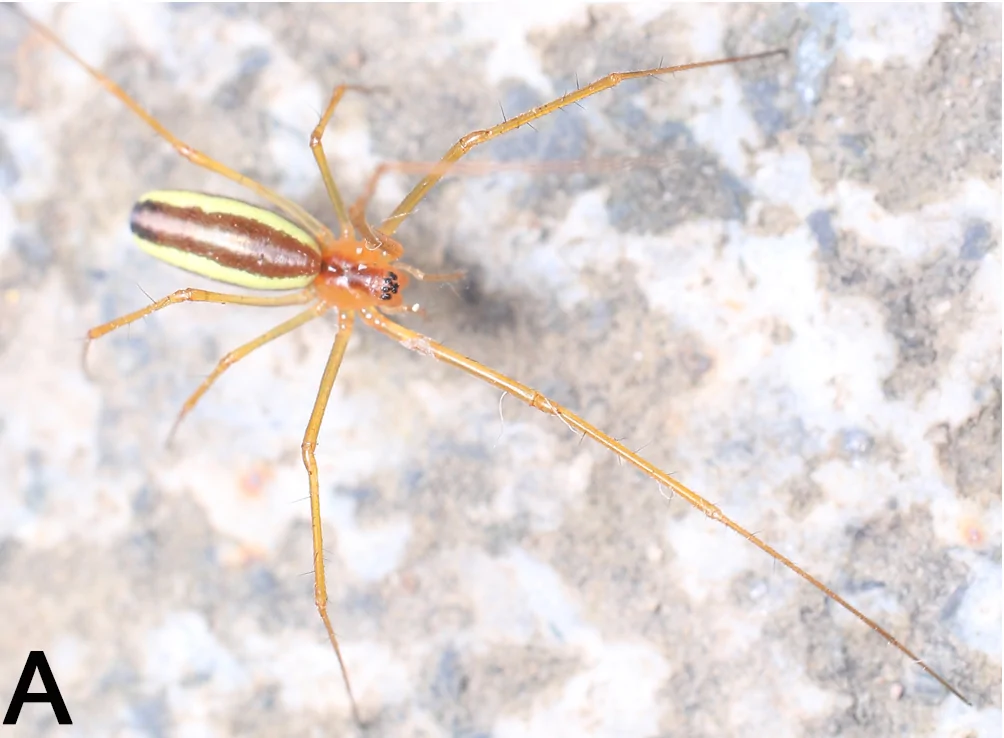


**Figure 2b.**
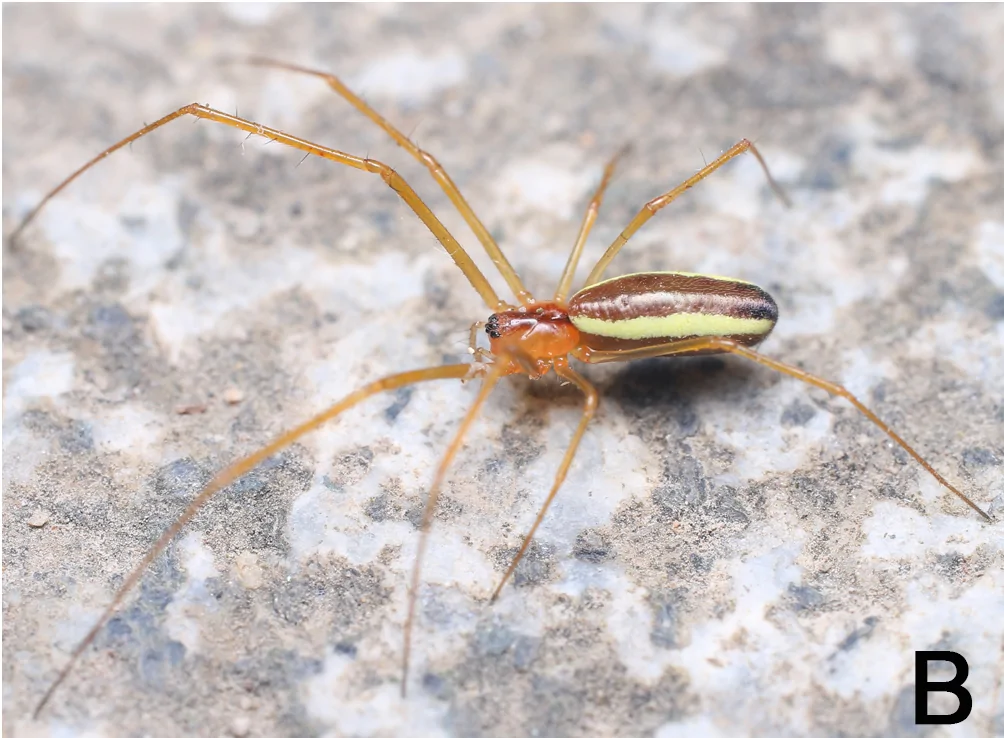


**Figure 2c.**
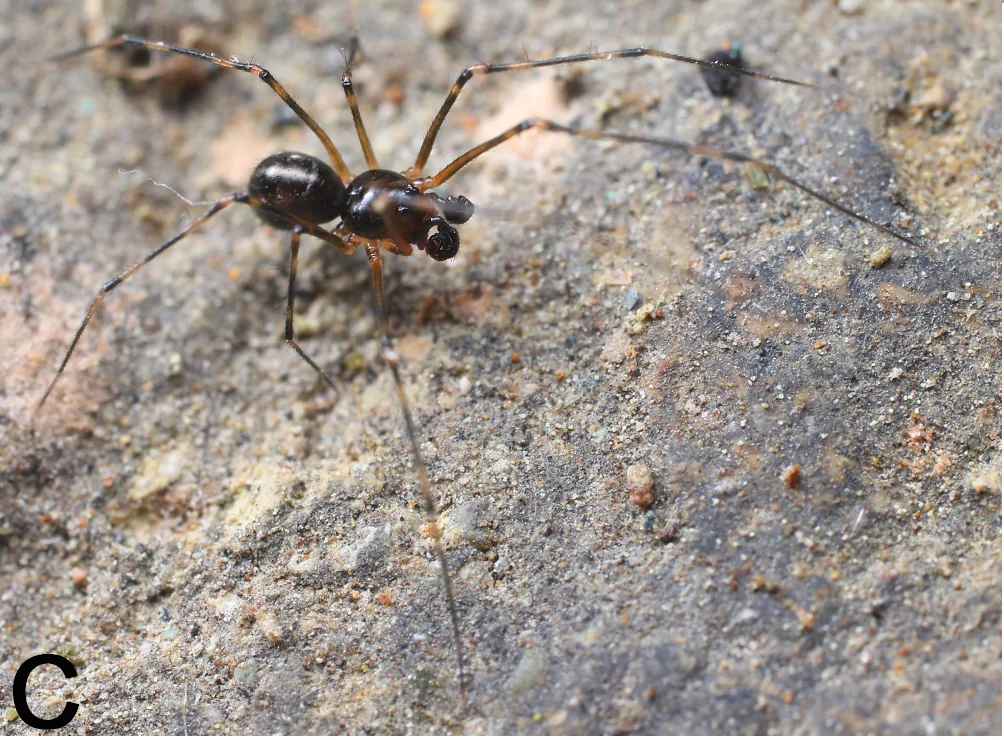


**Figure 2d.**
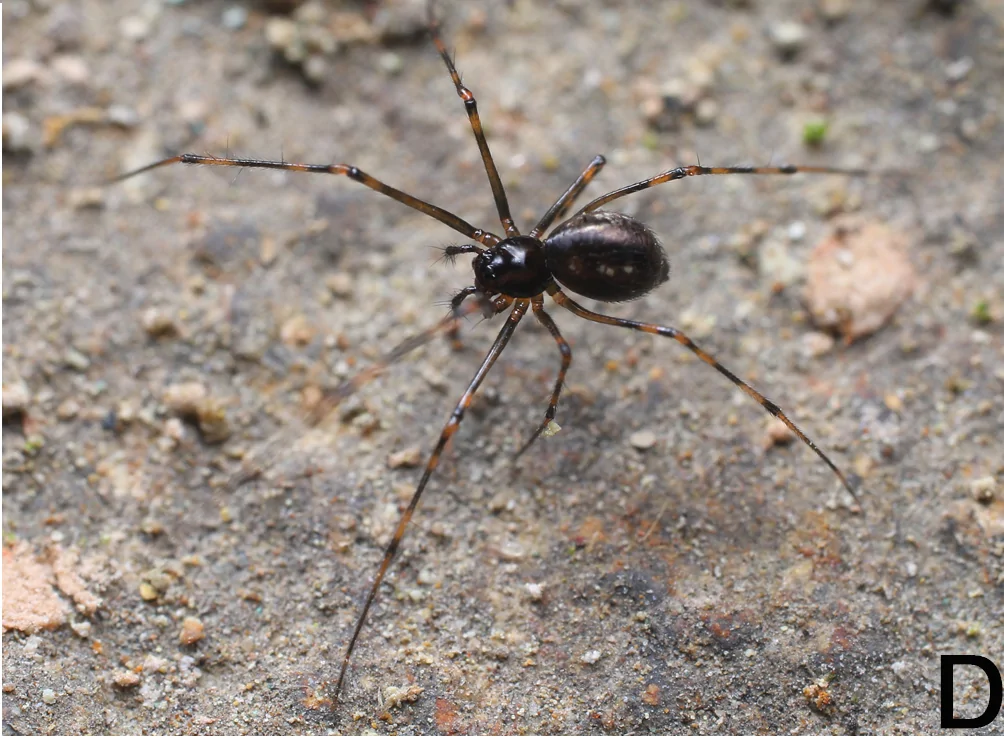


**Figure 2e.**
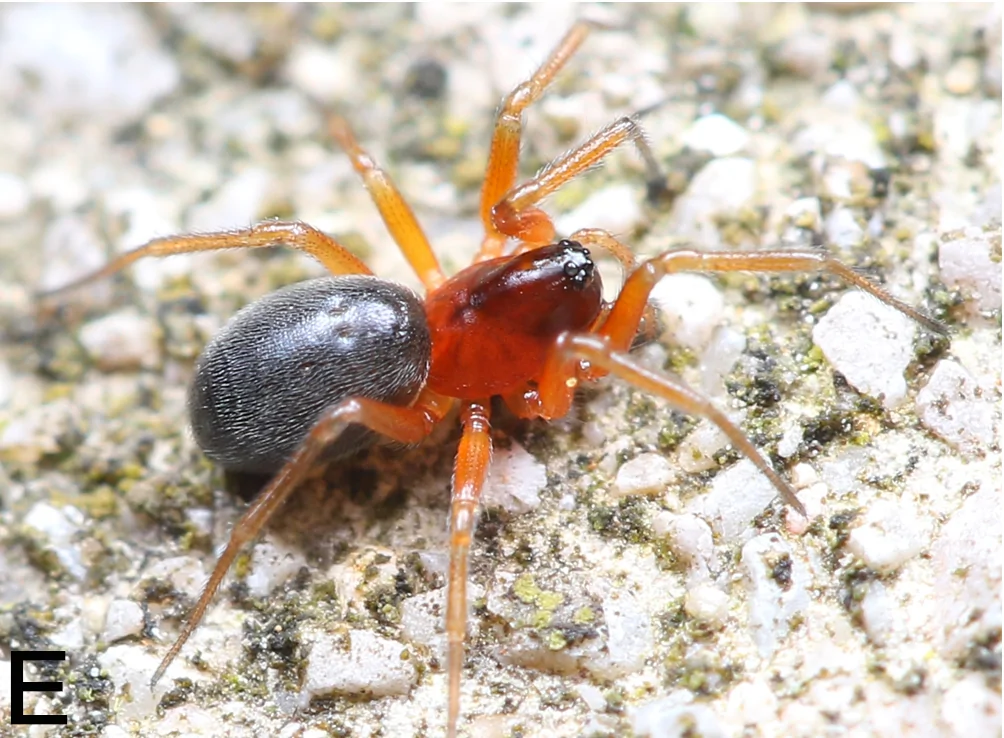


**Figure 2f.**
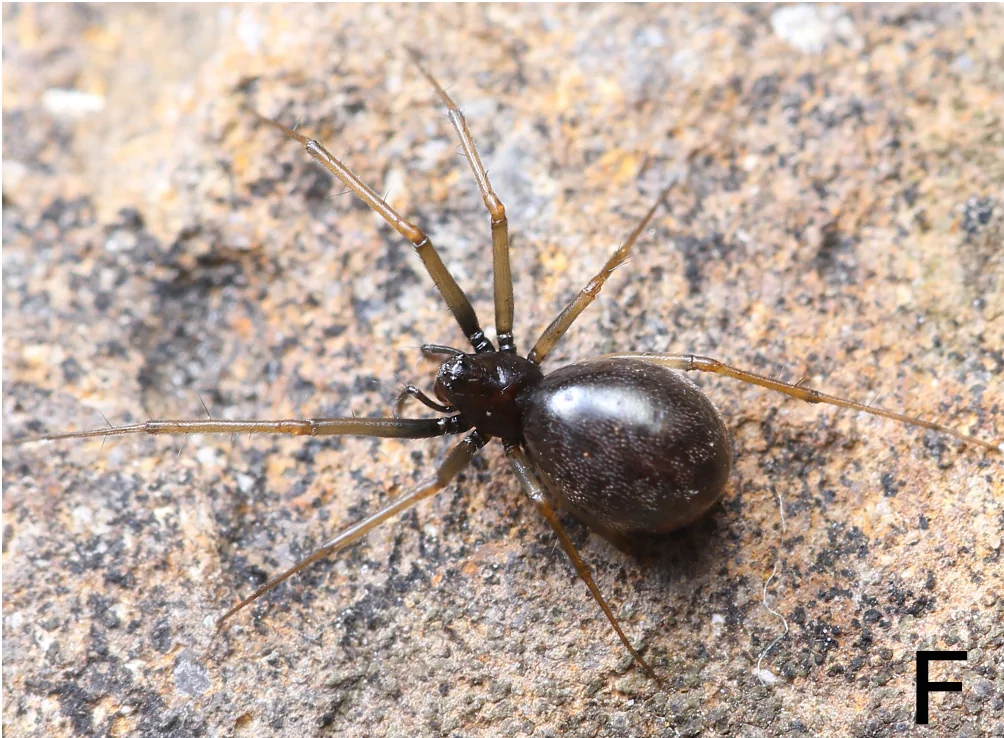


**Figure 3a.**
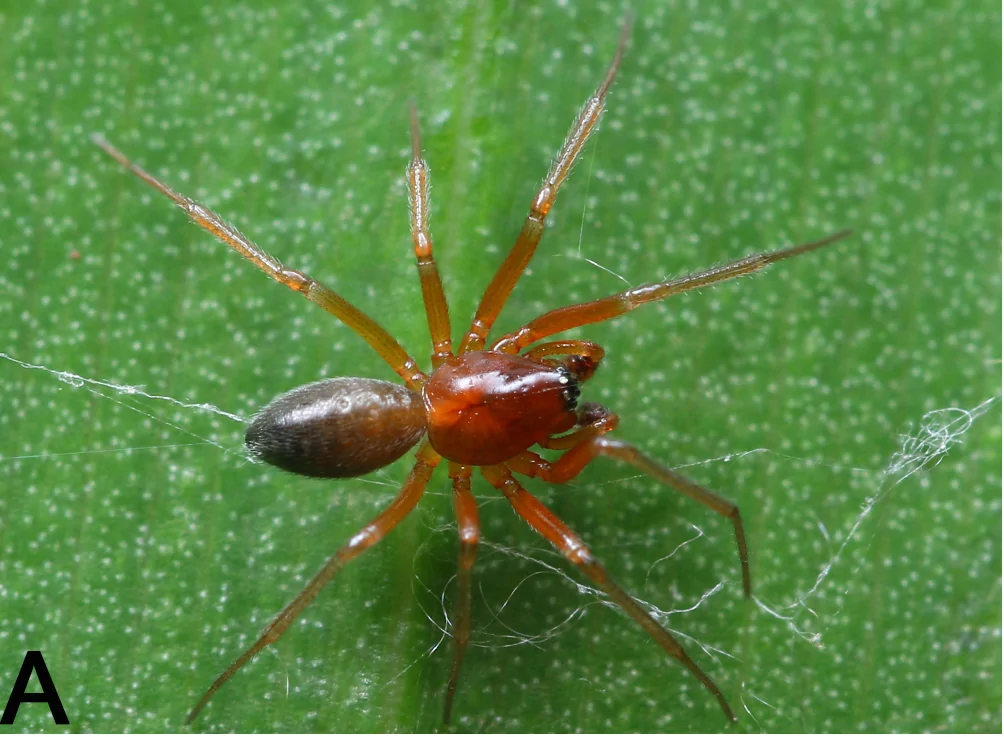


**Figure 3b.**
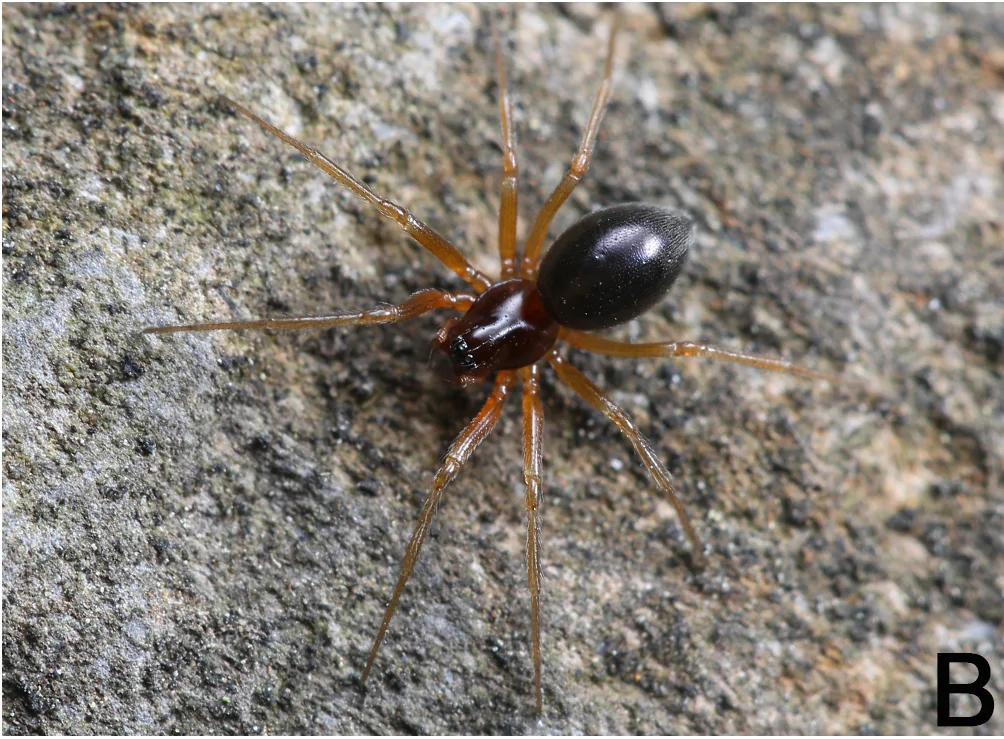


**Figure 3c.**
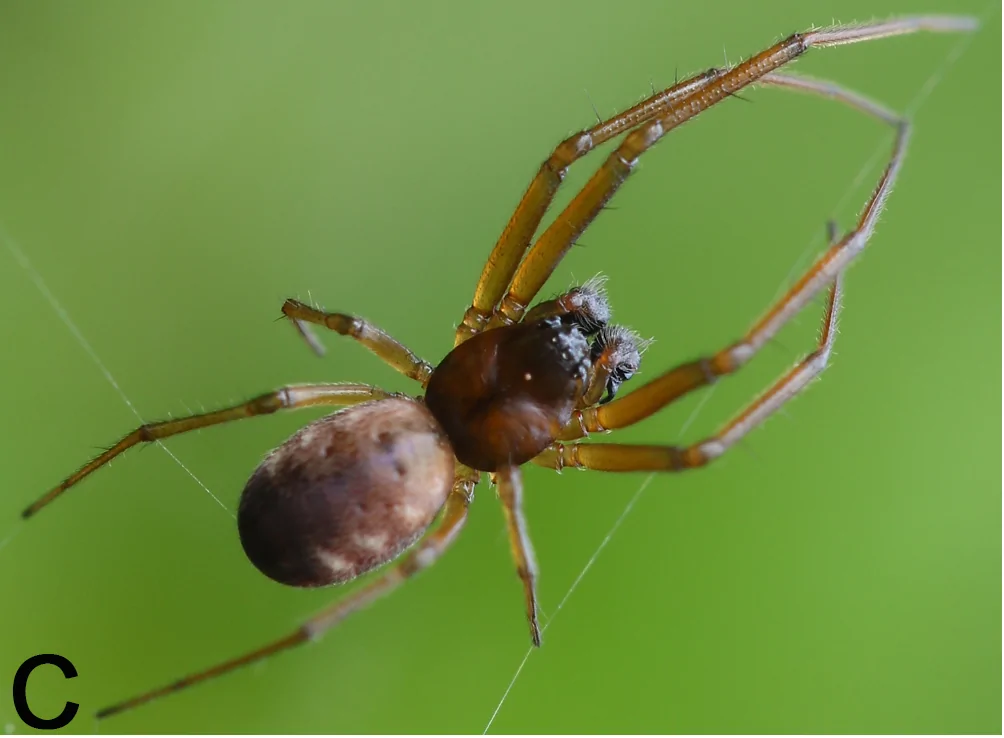


**Figure 3d.**
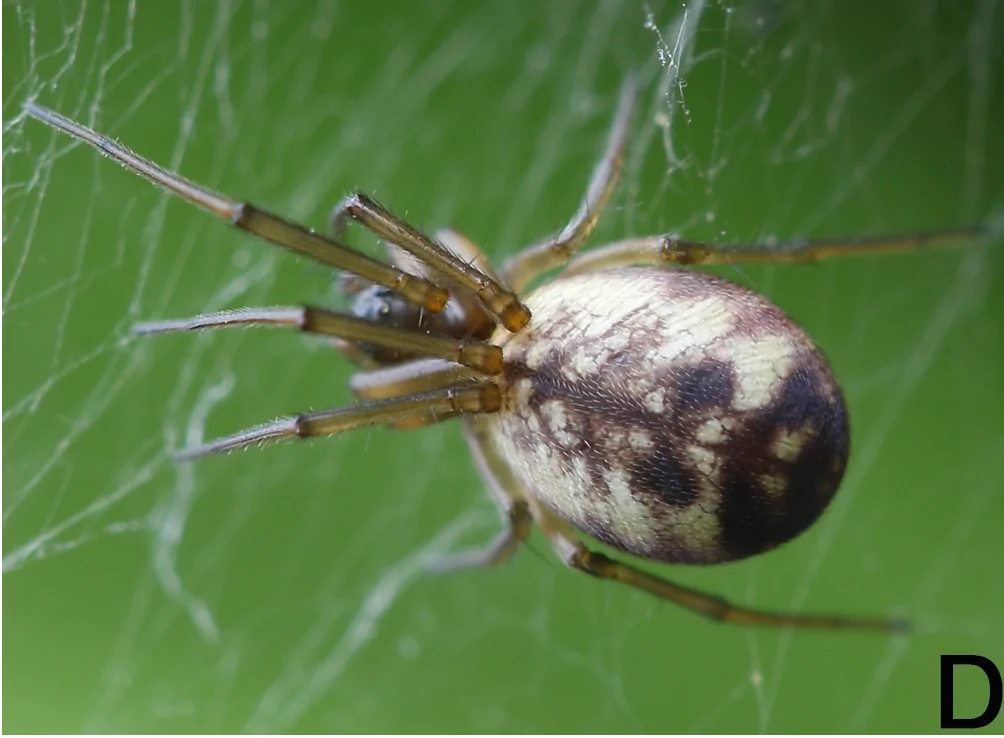


**Figure 3e.**
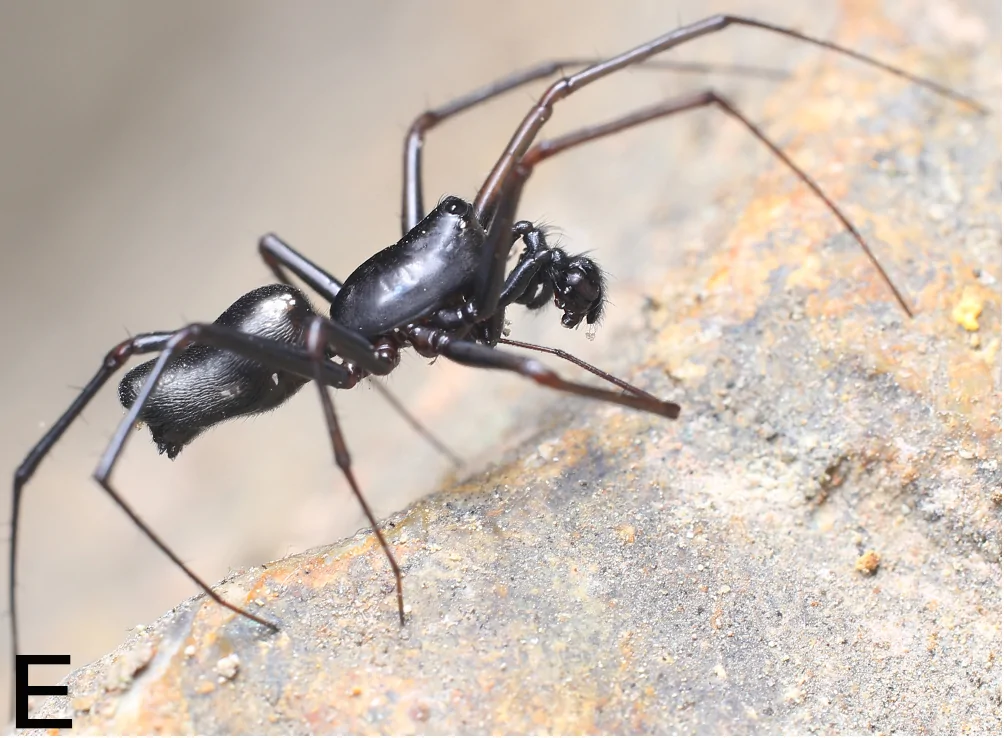


**Figure 3f.**
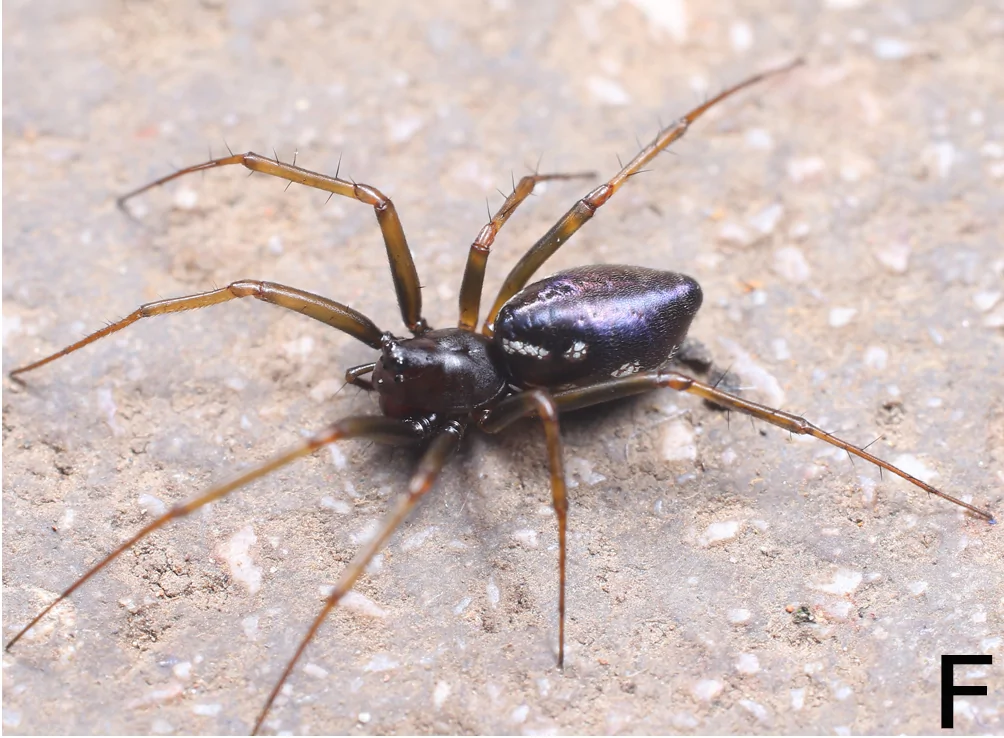


**Figure 4a.**
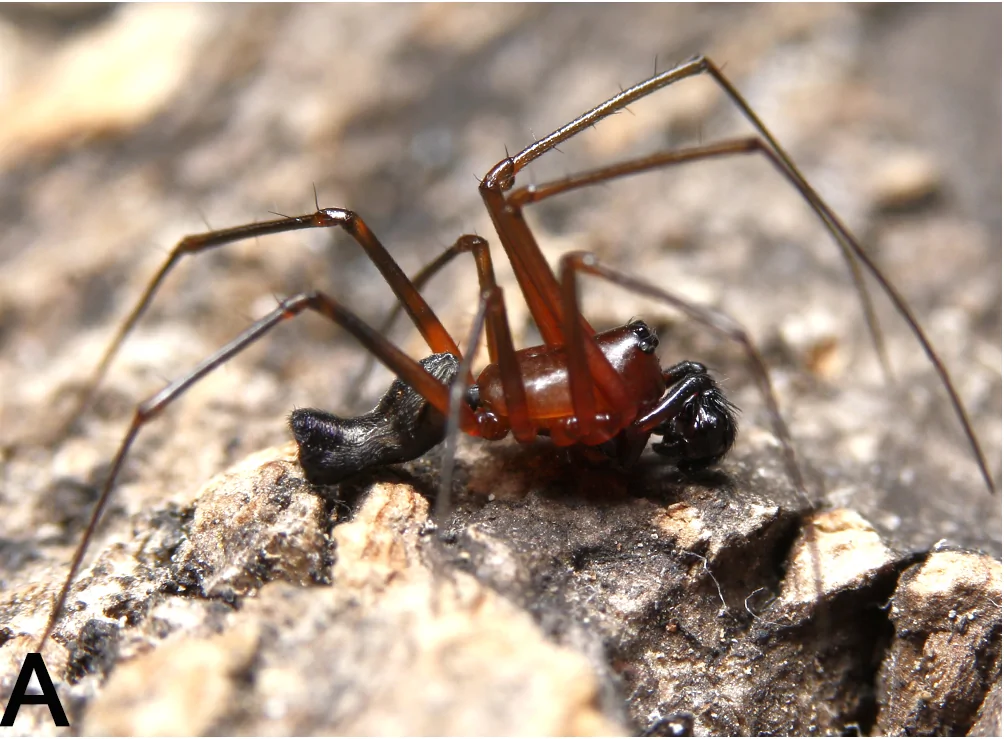


**Figure 4b.**
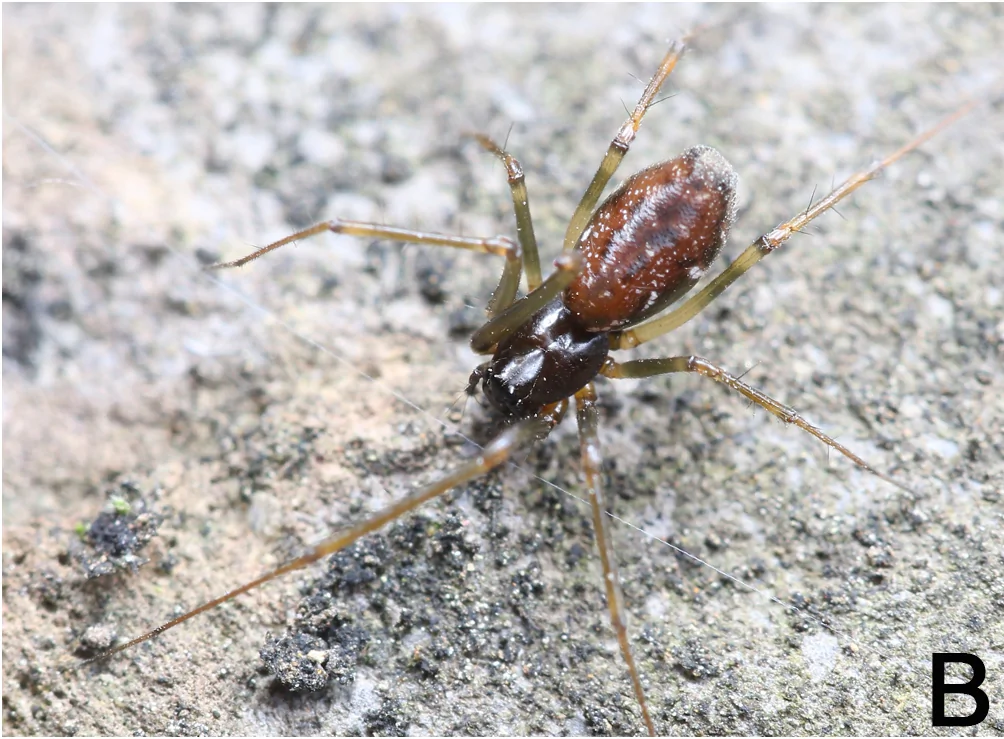


**Figure 4c.**
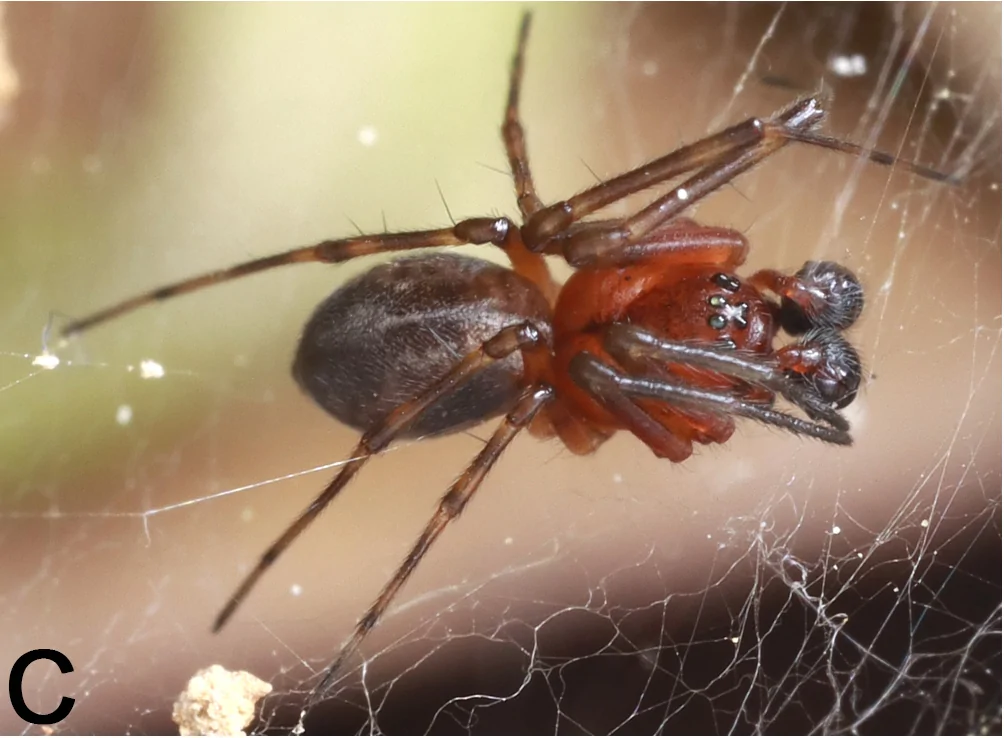


**Figure 4d.**
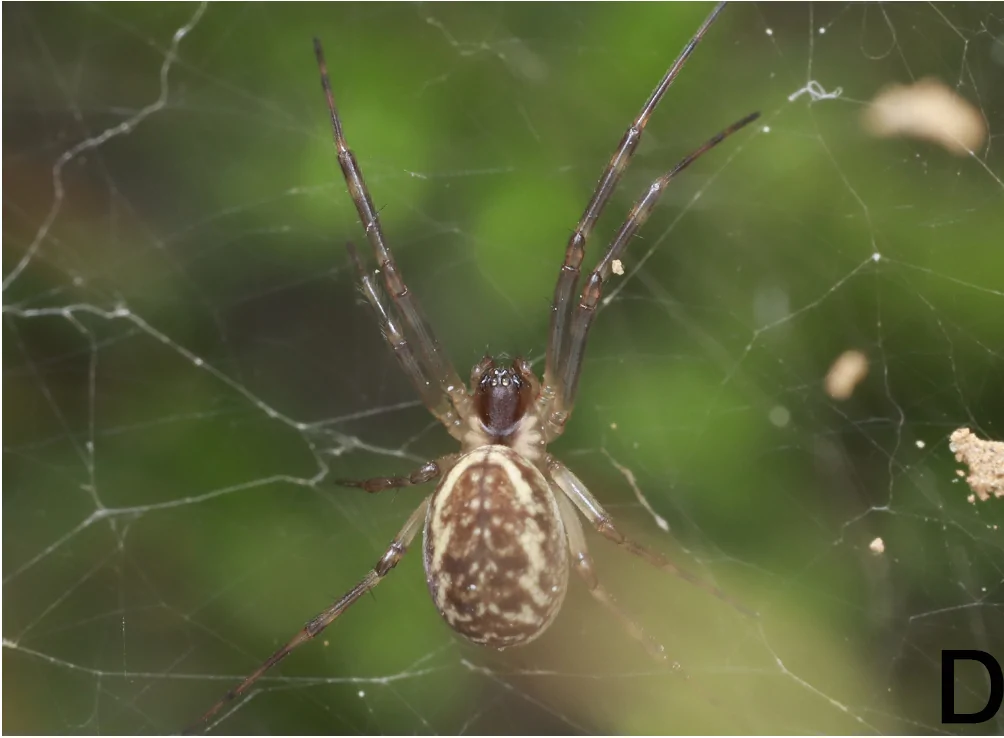


**Figure 4e.**
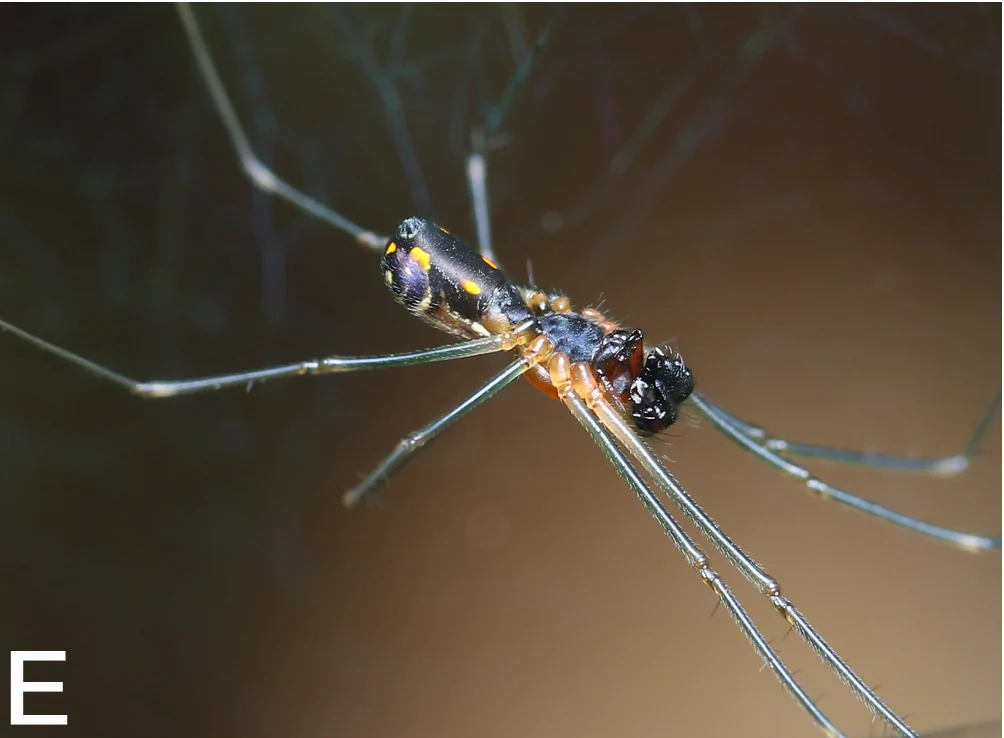


**Figure 4f.**
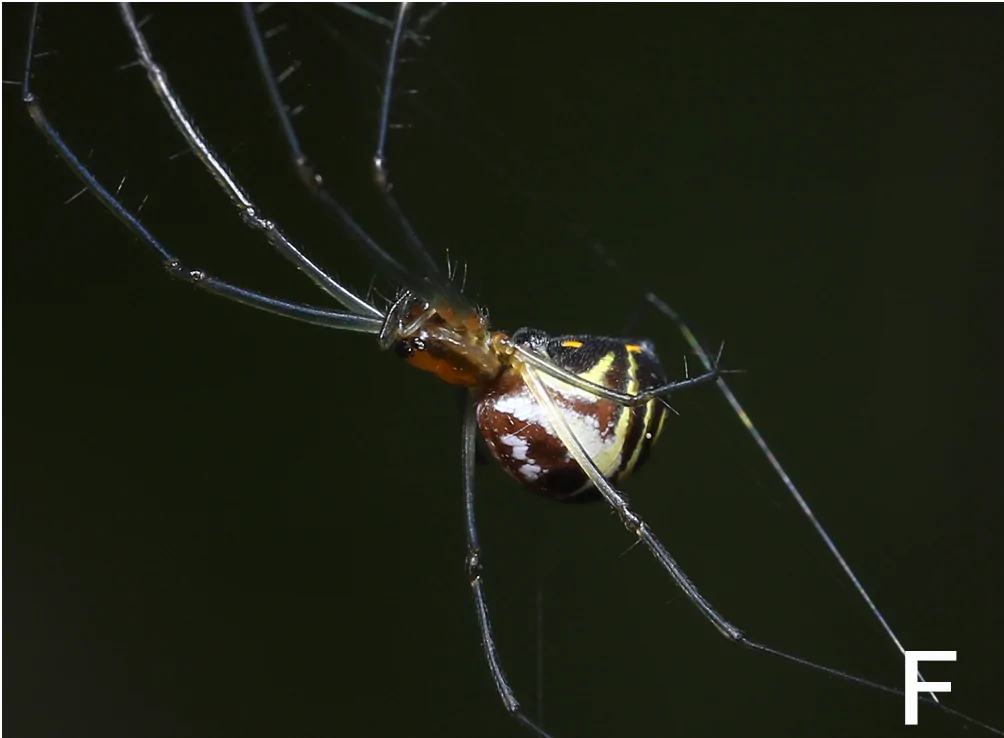


**Figure 5a.**
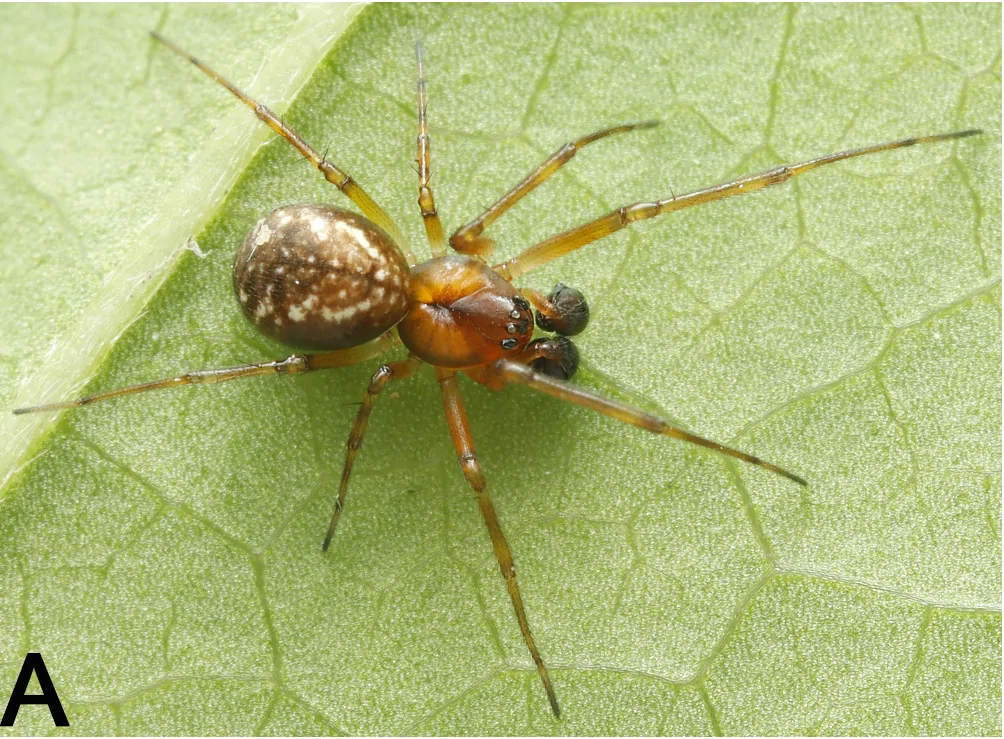


**Figure 5b.**
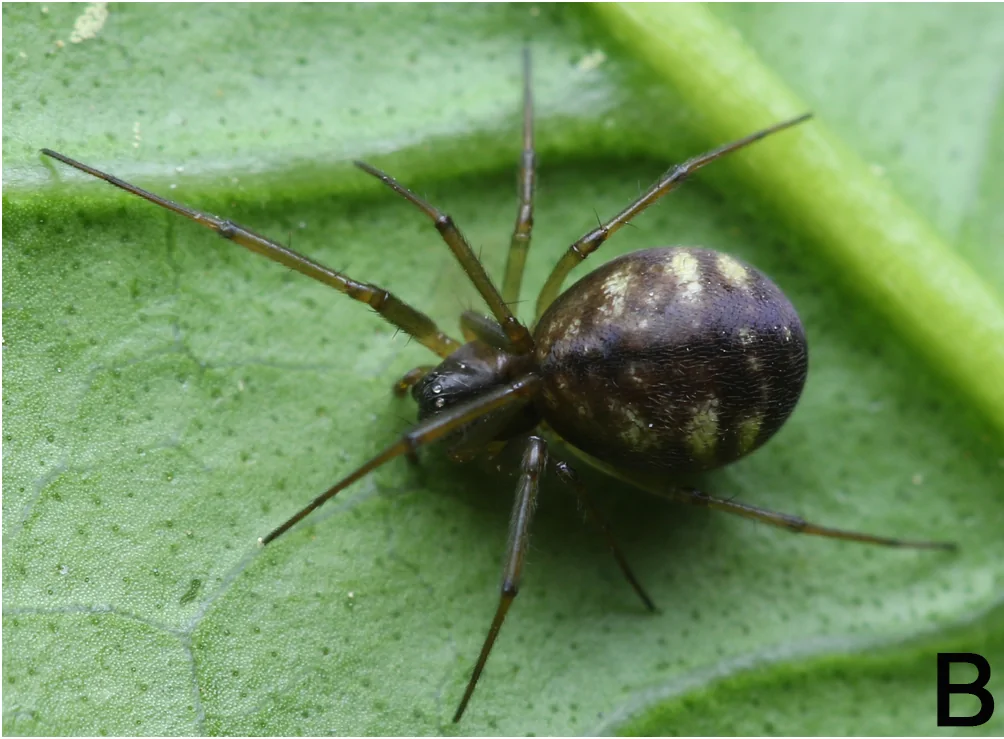


**Figure 5c.**
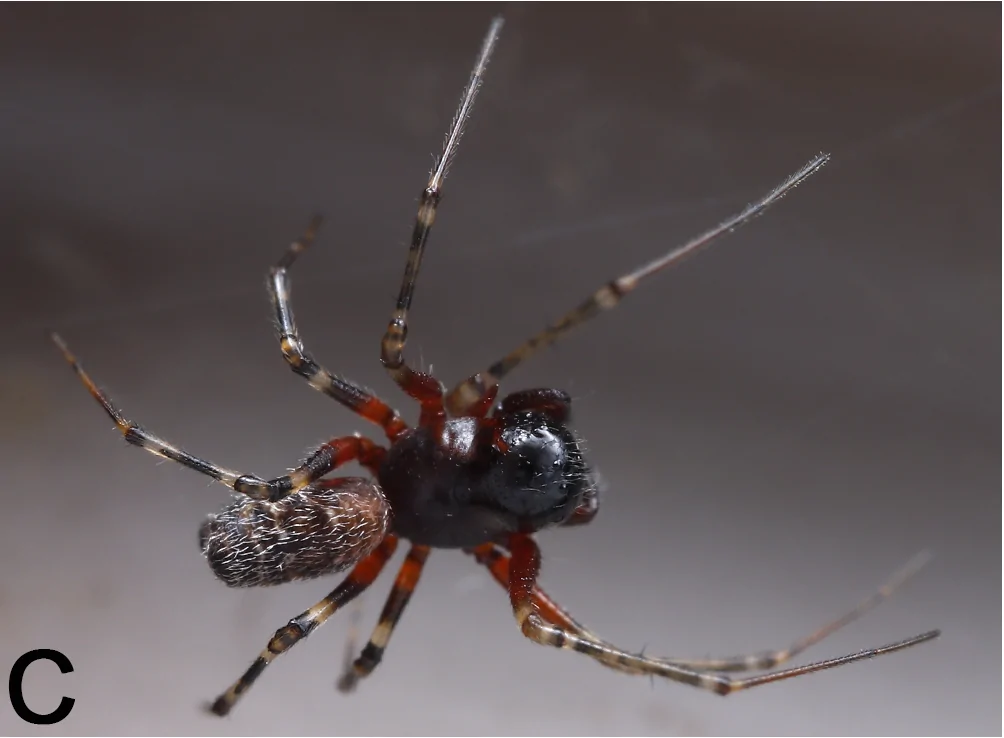


**Figure 5d.**
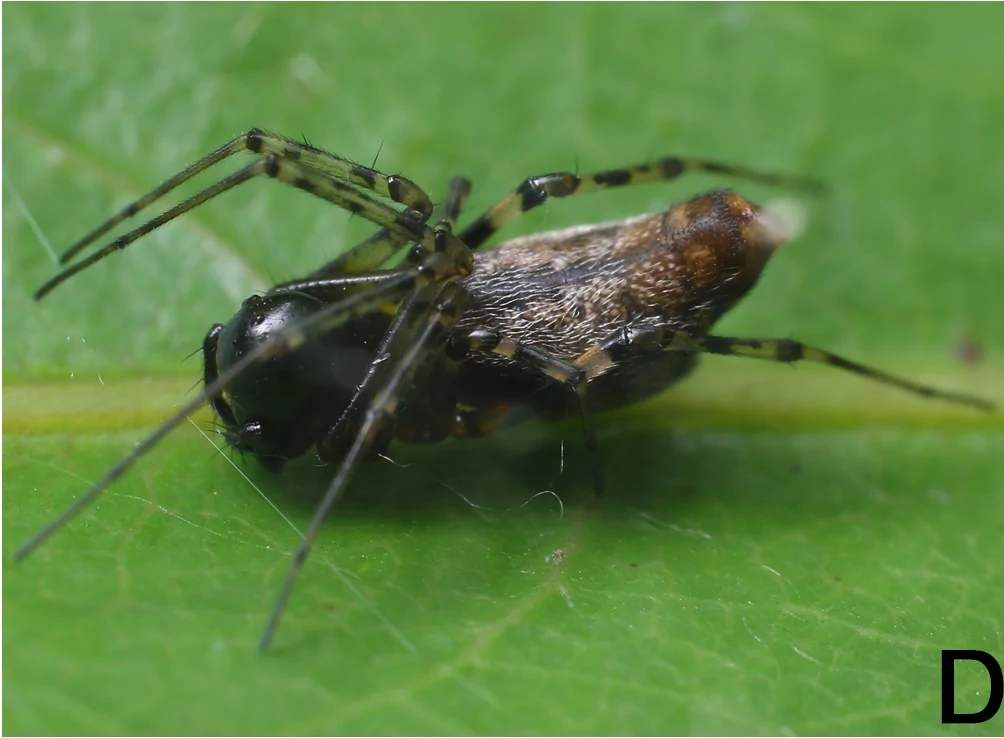


**Figure 5e.**
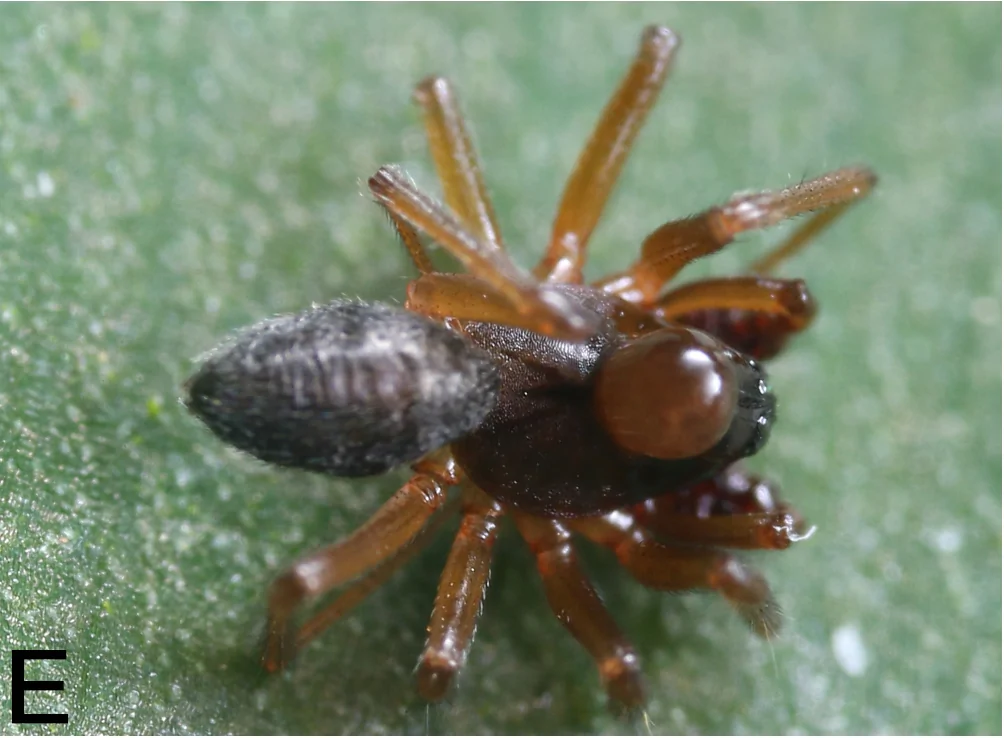


**Figure 5f.**
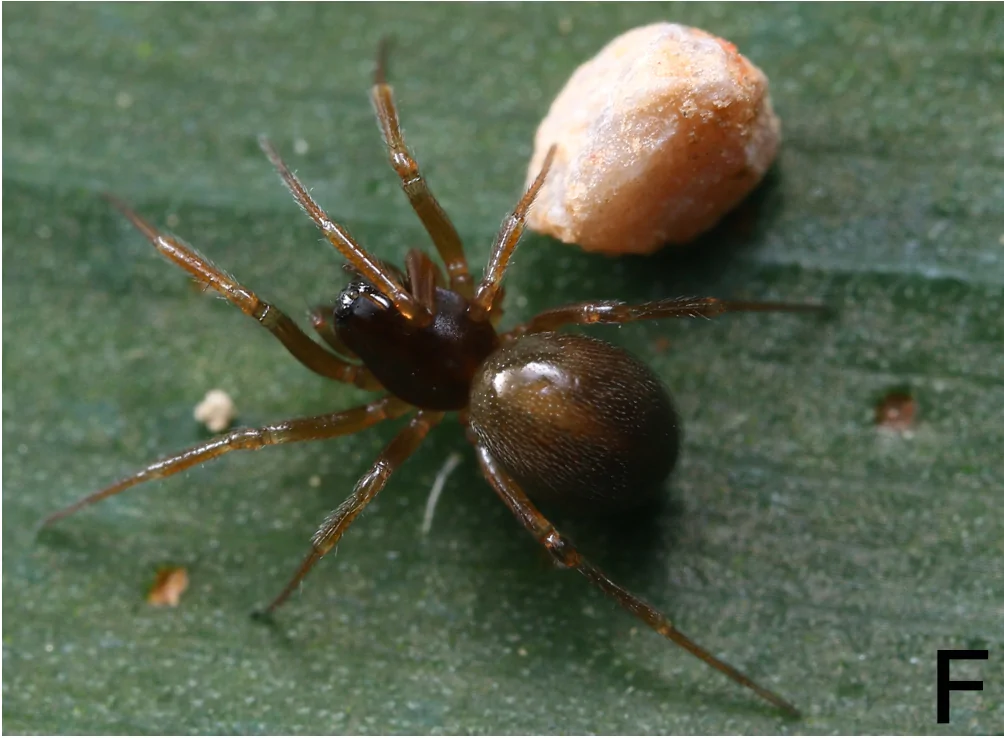


**Figure 6.**
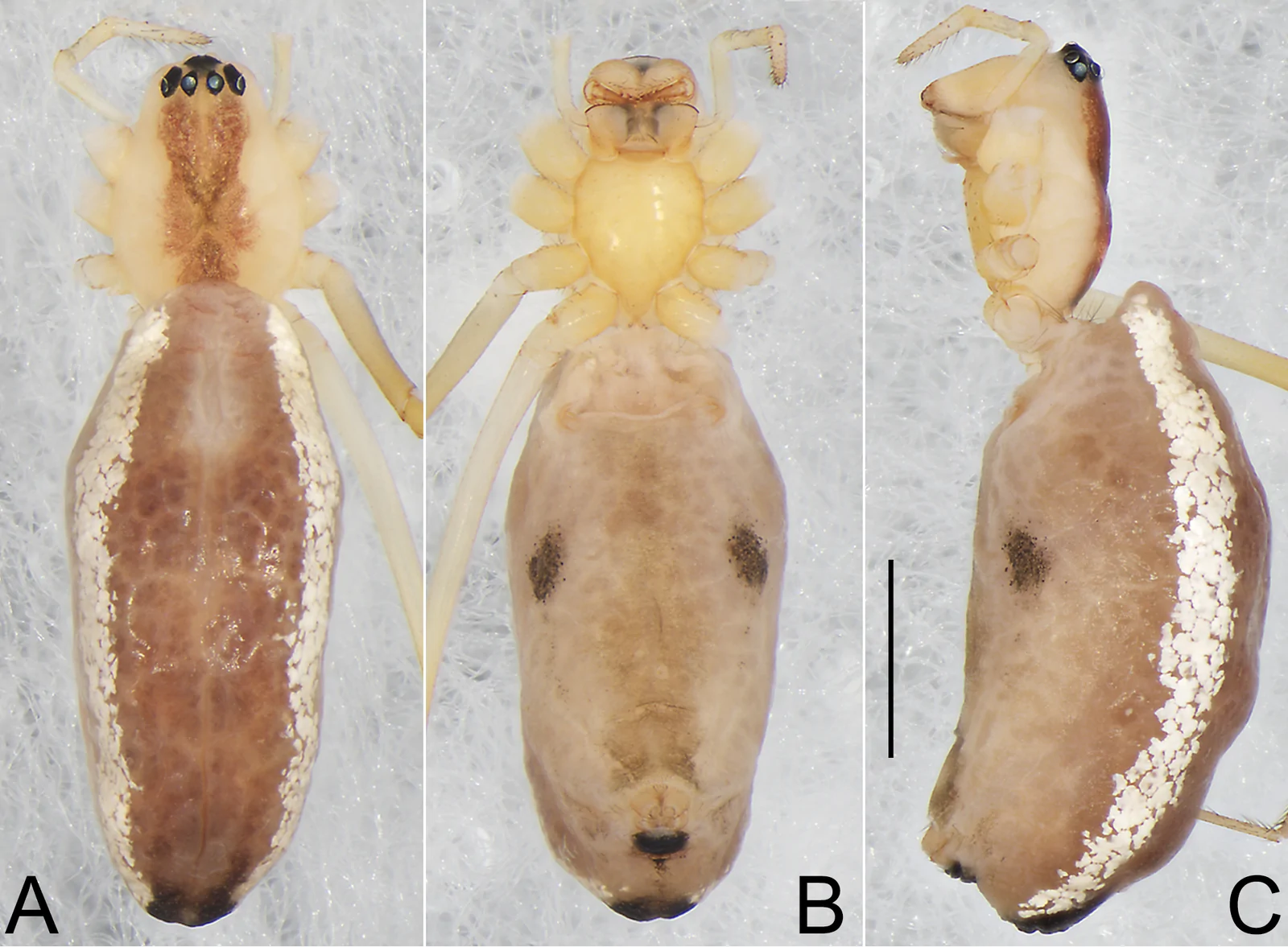
Habitus of the female holotype of *Lenis guiyang* He, Yu & Zhang, sp. nov. **A** dorsal view; **B** ventral view; **C** lateral view. Scale bar: 1 mm (equal for **A–C**).

**Figure 7.**
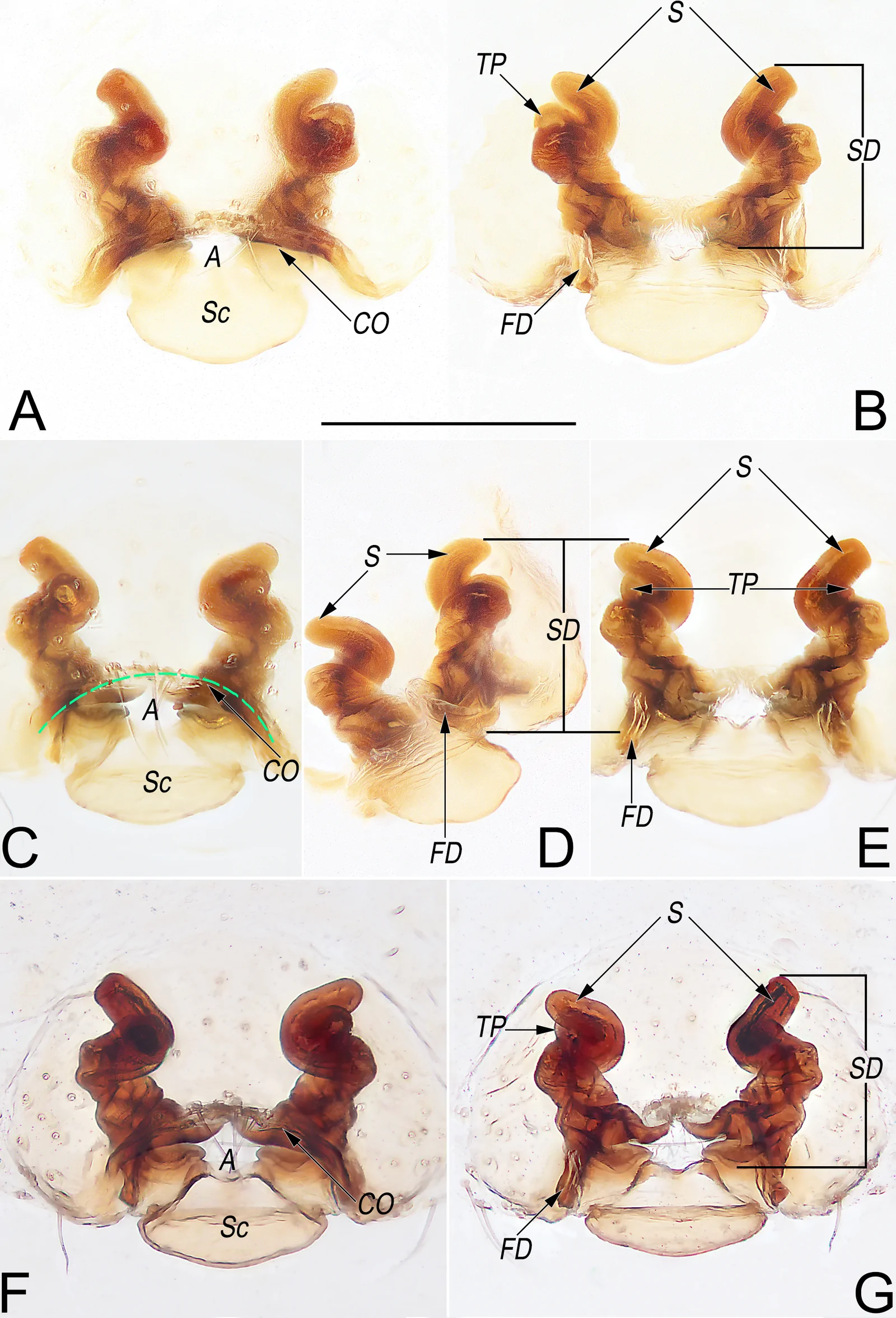
Epigyne of the female holotype of *Lenis guiyang* He, Yu & Zhang, sp. nov., macerated epigyne (**A–E**) and macerated epigyne embedded in Arabic gum (**F, G**). **A** anterior-ventral view; **B** posterior-dorsal view; **C, F** ventral view; **D** lateral view; **E, G** dorsal view. Abbreviations: A = atrium (green dashed line showing the anterior margin of the atrium); CO = copulatory opening; FD = fertilisation duct; S = spermatheca; Sc = scape; SD = spiral duct; TP = turning point. Scale bar: 0.2 mm (equal for **A–G**).
